# The HDAC7–TET2 epigenetic axis is essential during early B lymphocyte development

**DOI:** 10.1093/nar/gkac619

**Published:** 2022-07-29

**Authors:** Alba Azagra, Ainara Meler, Oriol de Barrios, Laureano Tomás-Daza, Olga Collazo, Beatriz Monterde, Mireia Obiols, Llorenç Rovirosa, Maria Vila-Casadesús, Mónica Cabrera-Pasadas, Mar Gusi-Vives, Thomas Graf, Ignacio Varela, José Luis Sardina, Biola M Javierre, Maribel Parra

**Affiliations:** Lymphocyte Development and Disease Group, Josep Carreras Leukaemia Research Institute, 08916 Badalona, Spain; Cellular Differentiation Group, Cancer Epigenetics and Biology Program (PEBC), Bellvitge Biomedical Research Institute (IDIBELL), Av. Gran Via 199, 08908 L’Hospitalet, Barcelona, Spain; Lymphocyte Development and Disease Group, Josep Carreras Leukaemia Research Institute, 08916 Badalona, Spain; Cellular Differentiation Group, Cancer Epigenetics and Biology Program (PEBC), Bellvitge Biomedical Research Institute (IDIBELL), Av. Gran Via 199, 08908 L’Hospitalet, Barcelona, Spain; Lymphocyte Development and Disease Group, Josep Carreras Leukaemia Research Institute, 08916 Badalona, Spain; Cellular Differentiation Group, Cancer Epigenetics and Biology Program (PEBC), Bellvitge Biomedical Research Institute (IDIBELL), Av. Gran Via 199, 08908 L’Hospitalet, Barcelona, Spain; 3D Chromatin Organization Group, Josep Carreras Leukaemia Research Institute, 08916 Badalona, Spain; Barcelona Supercomputing Center (BSC), Barcelona, Spain; Lymphocyte Development and Disease Group, Josep Carreras Leukaemia Research Institute, 08916 Badalona, Spain; Cellular Differentiation Group, Cancer Epigenetics and Biology Program (PEBC), Bellvitge Biomedical Research Institute (IDIBELL), Av. Gran Via 199, 08908 L’Hospitalet, Barcelona, Spain; Instituto de Biomedicina y Biotecnología de Cantabria. Universidad de Cantabria-CSIC. 39011 Santander, Spain; Epigenetic Control of Haematopoiesis Group, Josep Carreras Leukaemia Research Institute, 08916 Badalona, Spain; 3D Chromatin Organization Group, Josep Carreras Leukaemia Research Institute, 08916 Badalona, Spain; Centre for Genomic Regulation (CRG), PRBB Building, Dr. Aiguader 88, 08003 Barcelona, Spain; 3D Chromatin Organization Group, Josep Carreras Leukaemia Research Institute, 08916 Badalona, Spain; Barcelona Supercomputing Center (BSC), Barcelona, Spain; Lymphocyte Development and Disease Group, Josep Carreras Leukaemia Research Institute, 08916 Badalona, Spain; Centre for Genomic Regulation (CRG), PRBB Building, Dr. Aiguader 88, 08003 Barcelona, Spain; Universitat Pompeu Fabra, Barcelona, Spain; Instituto de Biomedicina y Biotecnología de Cantabria. Universidad de Cantabria-CSIC. 39011 Santander, Spain; Epigenetic Control of Haematopoiesis Group, Josep Carreras Leukaemia Research Institute, 08916 Badalona, Spain; 3D Chromatin Organization Group, Josep Carreras Leukaemia Research Institute, 08916 Badalona, Spain; Lymphocyte Development and Disease Group, Josep Carreras Leukaemia Research Institute, 08916 Badalona, Spain; Cellular Differentiation Group, Cancer Epigenetics and Biology Program (PEBC), Bellvitge Biomedical Research Institute (IDIBELL), Av. Gran Via 199, 08908 L’Hospitalet, Barcelona, Spain

## Abstract

Correct B cell identity at each stage of cellular differentiation during B lymphocyte development is critically dependent on a tightly controlled epigenomic landscape. We previously identified HDAC7 as an essential regulator of early B cell development and its absence leads to a drastic block at the pro-B to pre-B cell transition. More recently, we demonstrated that HDAC7 loss in pro-B-ALL in infants associates with a worse prognosis. Here we delineate the molecular mechanisms by which HDAC7 modulates early B cell development. We find that HDAC7 deficiency drives global chromatin de-condensation, histone marks deposition and deregulates other epigenetic regulators and mobile elements. Specifically, the absence of HDAC7 induces TET2 expression, which promotes DNA 5-hydroxymethylation and chromatin de-condensation. HDAC7 deficiency also results in the aberrant expression of microRNAs and LINE-1 transposable elements. These findings shed light on the mechanisms by which HDAC7 loss or misregulation may lead to B cell–based hematological malignancies.

## INTRODUCTION

A longstanding fundamental question in the field of cell development has been: how do cells decide at a molecular level to acquire a specific cell fate during tissue and organ generation? The mammalian hematopoietic system is considered a paradigm model for answering this question. For instance, B cell lymphopoiesis is a complex developmental process that comprises several cellular transitions, including cell commitment and early and late cellular differentiation. Proper transcriptional control at each cellular transition is essential for the correct generation of B lymphocytes. Of note, aberrant establishment of specific transcriptional programs may lead to the development of B cell malignancies.

Lineage-specific networks of transcription factors (TFs) have a central role in positively regulating the transition and maintenance of the distinct B cell developmental stages. In the bone marrow, lymphoid-primed multipotent progenitors (LMPPs) have the capacity and plasticity to become either common lymphoid progenitors (CLPs) or common myeloid progenitors (CMPs) (1). At that stage, the TFs IKAROS, MEF2C, and PU.1 are crucial for the early cellular choice towards the lymphoid lineage (2,3). Later on, commitment to the B cell lineage from CLPs to B cell progenitors (pro-B) and B cell precursors (pre-B) depends on the hierarchical and coordinated actions of the TFs PAX5, E2A, EBF and MEF2C (4–6). Classically thought as genuine activators for specific gene expression, B cell TFs are now believed to be involved in the repression of inappropriate lineage or of functionally undesirable gene transcription, thereby ensuring that the proper B cell identity and differentiation are maintained. In addition to positively promoting B lymphocyte gene-specific programs, the PAX5, E2A, EBF1 and MEF2C TFs induce the repression of inappropriate genes of alternative lineages, thereby ensuring maintenance of proper B cell identity and differentiation (3,7–11). These findings support the concept of transcriptional repression as an essential mechanism for proper B lymphocyte generation. However, the identity of master transcriptional repressors essential for establishing and maintaining the genetic identity of B lymphocytes has remained elusive for many years.

Besides specific TF networks, B cell differentiation also requires that epigenetic regulators and architectural proteins establish the correct permissive/non-permissive chromatin structure (euchromatin/heterochromatin) (12). There is a close relationship between transcriptional regulators and dynamic changes in the DNA epigenetic landscape during B cell development, with DNA methylation levels and the chromatin conformation state dynamically changing at every differentiation cell stage, giving them a specific epigenetic signature (13–17). Also, TFs such as PAX5 interact with architectural proteins that mediate long-range chromatin interactions (18). In mammals, DNA demethylation depends on the action of the Ten-Eleven Translocation (TET) enzyme family of TET1, TET2 and TET3, which convert 5-methylcytosine (5-mC) to 5-hydroxymethylcytosine (5-hmC), leading to DNA demethylation and consequent gene expression (19). Notably, TET2 has been shown to play crucial roles during hematopoiesis (20–22). Although broadly expressed within the hematopoietic system, myeloid cells express higher levels of TET2 compared with lymphoid cell populations (23–25). To date, the molecular mechanisms controlling different TET2 physiological levels within the hematopoietic systems are largely unknown.

We have previously reported that HDAC7 is a master transcriptional repressor in early B cell development, controlling the expression of lineage-inappropriate genes and thus the identity of pro-B cells. A lack of HDAC7 in pro-B cells leads to a block in B cell differentiation, aberrant activation of alternative lineage genes, a reduction of proliferation, and an increase in apoptosis (26). More recently, we have identified HDAC7 as a prognostic factor and biomarker of survival in infants with pro-B acute lymphoblastic leukemia (pro-B-ALL) and MLL-AF4 rearrangement, who display a general loss in HDAC7 expression; notably, the lowest levels of HDAC7 are associated with the poorest outcome for the infants (27). We hypothesized that these findings could be indicative of a yet-unknown HDAC7-mediated molecular mechanisms that allows proper acquisition of cell identity during early B cell development in the bone marrow.

Using a combination of transcriptomic and epigenetic genome-wide analysis, we now shed light into the molecular mechanisms that are governed by HDAC7 during early B cell development. We identified HDAC7 as a regulator of proper chromatin compaction in different stages of B cell development (pro-B and pre-B cells). Importantly, we demonstrated that HDAC7 represses TET2 expression in pro-B and pre-B cells, and that its deficiency leads to TET2 up-regulation and subsequent alteration in global and specific 5-hmC patterns. In fact, HDAC7*-*deficient pro-B cells showed enhanced 5-hmC global levels, resulting not only in the activation of inappropriate lineage genes, but also in the aberrant expression of non-coding elements (such as active transposon LINE-1 elements and miRNAs). Thus, our findings unveil novel molecular mechanisms that govern the maintenance of correct B cell development and identity, working through the HDAC7–TET2 axis.

## MATERIALS AND METHODS

### Study Design

The study aimed to define unprecedented molecular mechanisms by which class IIa HDAC7 preserves B cell identity in mice. Experiments included 4–6 weeks-old wild-type and knockout mouse strains (C57BL/6). Mice selected in each experiment were littermates. Primary pro-B and pre-B lymphocytes were isolated by using cell sorting. Tet2 was identified as a direct target of HDAC7 with chromatin immunoprecipitation and expression analysis experiments. 5-hydroxymethylation levels in pro-B cells were quantified by ELISA and hydroxymethyl-DIP experiments. 5-hmeDIP-seq, ATAC-seq, H3K27ac and H3K27me3 ChIP-seq were performed to determine global and specific changes. All results were validated by qPCR assays and were successfully reproduced. No sample size calculations were performed, since these were selected on the basis of previous studies performed in our lab. The numbers of experimental replicates are included in the figure legends.

### Mice

*Hdac7^fl/−^* on C57BL/6 mice have been previously described (28) and were kindly provided by Dr Eric Olson (UT Southwestern Medical Center, Dallas, TX, USA). Mb1-Cre^ki/+^ on C57BL/6 (B6.C(Cg)-Cd79a^tm1(cre)Reth^/EhobJ) mice were kindly provided by Dr Michael Reth (Max Planck Institute of Immunology and Epigenetics, Freiburg, Germany). Experiments were performed with 4–6 week-old mice. Littermate controls were used for *Hdac7^fl/^*^−^ mb1-cre mice. Animal housing and handling and all procedures involving mice, were approved by the Bellvitge Biomedical Research Institute (IDIBELL) ethics committee and the Animal Experimentation Ethics Committee (CEEA) of the Comparative Medicine and Bioimage Centre of Catalonia (CMCiB), at Germans Trias i Pujol Research Institute (IGTP), in accordance with Spanish national guidelines and regulations.

### Cells

HAFTL pre-B cell line transduced with a MSCV-GFP-C/EBPα retroviral vector (to generate C10 cells) and with a MSCV-hCD4-C/EBPαER retroviral vector (to generate C11 cells), were described previously described (29). C10-MSCV and C10-HDAC7 cells were generated as previously described (30).

### shRNA-Tet2 infection of primary B cells and transduced pre-B cell line (C11)

Retroviral vector for PGK-shRNATet2-GFP retroviral vector has been described in (31). CD19^+^ B cells from *Hdac7^+/^^−^* and *Hdac7^fl/^^−^* mice or C11 cells were infected with the shRNA Tet2 targeting vector(shTet2) or with an empty retroviral vector (shCtrl). Cells were infected twice, in a time gap of 24 h, and then, 48 h after second infection, GFP^+^ cells were sorted using a FACSAria™ Fusion cell sorter (BD Biosciences). After isolation, CD19^+^ B cells were cultured on RPMI media supplemented with 2% FBS, 0,03% Primatone RL (Sigma), 1 mM penicillin/streptomycin, 50 μM β-mercaptoethanol and 1% IL-7 (Peprotech), whereas C11 cells were cultured in RPMI media supplemented with 10% FBS and 1mM penicillin/streptomycin. *Tet2* knockdown was confirmed by qRT-PCR using SYBR green quantification.

### β-Estradiol treatment of cell lines

C10-MSCV, C10-HDAC7, C11-shCtrl and C11-shTet2 cells were cultured and treated as previously described (30).

### Flow cytometry analysis and cell-sorting

Cells were extracted from bone marrow (femur and tibia of both hind legs) of *Hdac7^+/^^−^* and *Hdac7^fl/−^* mice. Red blood cells were lysed with ACK lysis buffer. Cell counts were determined using a manual cell counter and Türk's staining to facilitate the counting of white cells nuclei. Isolated cells were incubated with anti-CD16/CD32 (2.4G2, Fc Block) (BD Bioscience) for 10 min on ice to reduce non-specific staining. The following antibodies were used for analysis (from BD Biosciences): anti-B220 (RA3-6B2), anti-CD43 (S7), anti-IgM (R6-60.2), anti-CD19 (1D3) and anti-Cd11b (M1/70). For Cd11b, Streptavidin-V50 (560797, BD Biosciences) was used as a secondary antibody. Cells were stained with primary antibodies for 30 min on ice in the dark. Cells were analyzed on a BDFACS Canto II (BD Biosciences) or sorted on BD FACSAria™ Fusion cell sorter (BD Biosciences). Data were analyzed using FlowJo software (Tree Star, Inc.). See Supplementary Figure S1A for sorting gating strategy (pro-B cells: IgM^−^, CD19^+^, B220^+^, CD43^+^; pre-B cells: IgM^−^, CD19^+^, B220^+^, CD43^−^).

### Magnetic cell separation (MACS)

Cells were extracted from bone marrow (femur and tibia of both hind legs) of *Hdac7^+/^^−^* and *Hdac7^fl/−^* mice. Red blood cells were lysed with ACK lysis buffer. Cell counts were determined using a manual cell counter and Türk's staining to facilitate the counting of white cells nuclei. Isolated cells were incubated with anti-CD16/CD32 (2.4G2, Fc Block) (BD Bioscience) for 10 min on ice to reduce non-specific staining. The following antibodies were used for separation (from Miltenyi Biotec): anti-CD19-Microbeads (mouse), anti-Cd11b biotin (M1/70, BD Biosciences), and Streptavidin-Microbeads. Samples were incubated for 20 min at 4°C in the dark. CD11b needed double incubation, first with anti-Cd11b and second with Streptavidin-beads. Samples were put into Ls columns (Miltenyi Biotec) to perform magnetic cell separation. After three washes, cell from positive fractions (CD19+ and Cd11b+) were kept for further experimentation.

### RNA-sequencing and analysis

Total RNA was extracted from HDAC7-deficient and control pro-B and pre-B cells in the Genomics facility of Institute for Research in Biomedicine (IRB) in Barcelona. Samples were quantified and subjected to quality control using a Bioanalyzer apparatus (IRB, Barcelona). Samples were processed at BGI Genomics Service, (China). Briefly, low input library was performed in all samples. Later, they were sequenced in paired-end mode with a read length of 100 bp. Thirty-five million paired-end reads were generated for each sample. Quality control of the samples was performed with the FastQC tool (available at https://www.bioinformatics.babraham.ac.uk/projects/fastqc/). Paired-end reads from RNA-Seq were aligned to the murine reference genome (GRCm38) using Hisat2 (version 2.0.5). Quality of the alignments was assessed using FastQC (v0.11.2). A count table file indicating the number of reads per gene in each sample was generated using HTSeq (version 0.6.0) (32). Genes with no or very low expression were filtered out and differentially expressed genes were identified using DESeq (33), requiring a minimum adjusted *P*-value of 0.05 and a |log_2_FC| value >1. Functional analysis was performed using gene set enrichment analysis (GSEA) (34) using a pre-ranked list of human orthologs genes and the gene set database c5.all.v7.2.symbols.gmt (Gene Ontology). GSEA analyses were performed from the *Hdac7^+/^*^−^ pro-B versus *Hdac7^+/^^−^* pre-B cells, *Hdac7^+/−^* pro-B versus *Hdac7^fl/^^−^* pro-B cells and *Hdac7^+/^^−^* pro-B versus *Hdac7^fl/^^−^* pre-B cells comparisons. Genes were ranked using this formula: −log_10_(FDR) × sign[log(FC)]. As genesets collection, hallmarks (H) from the Molecular Signatures Database (MSigDB) were selected, adding the specified custom genesets. Data from RNA-seq is available under accession code GSE171855.

### RT-qPCR analysis

RNA from sorted pro-B and pre-B cells was extracted with an RNeasy Mini kit (Qiagen) and subsequently converted into cDNA using the High Capacity cDNA Reverse Transcription Kit (AB Applied Biosystems) according to the manufacturer's instructions. Real-time-quantitative PCR (RT-qPCR) was performed in triplicate using SYBR Green I Master (Roche). PCR reactions were run and analyzed using the LightCycler 480 Detection System (Roche). RT-qPCR primer pairs are shown in supplemental information Supplementary Table S1.

### Micrococcal nuclease assay

Two million of B cells from *Hdac7^+/^^−^* and *Hdac7^fl/^^−^* mice were resuspended in 500 μl of lysis buffer (10 mM Tris, 10 mM NaCl, 3 mM MgCl_2_, 1% Triton, pH 7.5) and incubated on ice for 10 min. Nuclei were collected by centrifugation at 300 g for 5 min at 4°C. The nuclear pellet was then resuspended in 400 μl of nuclear lysis buffer (20 mM Tris, 20 mM KCl, 70 mM NaCl, 3 mM CaCl_2_ and protease inhibitors, pH 7.5). Aliquots of 100 μl were incubated with 9 units of micrococcal nuclease (MNAse, ThermoFisher) and digested at room temperature for 0, 1, 2 and 5 min, respectively. Then 3μl of 0.5M EDTA was added to stop digestion, and DNA was purified by using the QIAquick PCR purification kit (Qiagen). About 500 ng were used for gel electrophoresis and 12 ng of DNA were used for qPCR analysis using SybrGreen (Roche). Primers obtained from (35) were designed to target the β-globin gene to obtain PCR amplicons longer than the length of a single nucleosome. Mononucleosomes generated during MNAse digestion cannot be amplified by qPCR. Then, reduced amplification involves more open/accessible chromatin state, at least at this locus.

### Western Blot

White cells from bone marrow of control and *Hdac7^fl/−^* mice were extracted as in sorting procedure. Next, cells were stained with anti-CD19-microBeads (Miltenyi Biotec) according to manufacturer's instructions. CD19^+^ B cells were isolated by magnetic separation with LS column adapters (Miltenyi Biotec). Purified cells were lysed with RIPA buffer. Lysates were resolved on 8–15% SDS-PAGE (Mini-Protean electrophoresis chamber, Bio-Rad) and transferred on nitrocellulose membranes (Amersham Biosciecnes). Membranes were blocked in 5% milk in TBS with 0.1% Tween (TBS-T) and incubated overnight at 4°C, with primary antibodies (anti-Tet2 ab94580, abcam 1:1000; anti-HDAC7 sc-11421, Santa Cruz Biotechnology 1:1000; anti-H3K9me3 ab8898 (Abcam) 1:1000; anti-PUMA 12450 (Cell Signaling) 1:500, anti-IRF4 sc-48338 (Santa Cruz Biotechnology) 1:500; anti-c-MYB sc-74512 (Santa Cruz Biotechnology) 1:500; anti-H3 ab1791 (Abcam) 1:1000; anti-Lamin B1 ab16048 (Abcam) 1:1000, and anti-Actin AC-15 (Sigma-Aldrich) 1:40000). Secondary antibody incubations (HRP-anti mouse, P0260, or anti rabbit, P0448, Dako 1:3000), were carried out for 1h at room temperature. Protein signal was detected using ECL western detection kit (Amersham Biosciences).

### Chromatin immunoprecipitation assays (ChIP)

For chromatin immunoprecipitation (ChIP) assays, purified pro-B cells from the bone marrow of *Hdac7*^+/−^ and *Hdac7*^fl/−^ mice were crosslinked for 15 min in 1% formaldehyde, followed by inactivation in 125 mM glycine for 5 min and by two washes in cold PBS. Afterward, samples were incubated in cell lysis buffer from LowCell# ChIP kit (Diagenode) for 30 min at 8°C and sonicated with M220-Focused Ultra Sonicator (Covaris) according to manufacturer's instructions. Next steps of ChIP experiments were performed using resources from the LowCell# ChIP kit (Diagenode) according to the manufacturer's instructions. The following antibodies were used for immunoprecipitation: anti-HDAC7 (Abcam, HDAC7-97, 2.5 μg), anti-H3K9/K14ac (Millipore, 06-599 2.5 μg), anti-H3K27me3 (Millipore, 07-449, 2.5 μg), anti-H3K27Ac (Abcam, ab4729, 2.5 μg) and anti-H3k9me3 (Abcam, ab8898, 2.5 μg). Real-time quantitative PCR (RT-qPCR) was performed in triplicate and the results analyzed. Data are presented as the ratio between the HDAC7-bound fraction and histone modification antibody relative to the input control. ChIP-qPCR primer pairs are shown in supplemental information Supplementary Table S1.

### ChIP-seq experiments and analysis

H3K9/K14ac ChIP-seq data were extracted from ChIP-seq experiments, as described elsewhere (26), whose data are available under the accession code: SRA submission SUB1614653. For new ChIP-seq experiments, purified pro-B and pre-B cells from the bone marrow of *Hdac7*^+/−^ and *Hdac7*^fl/−^ mice were crosslinked for 15 min in 1% formaldehyde, followed by inactivation in 125 mM glycine for 5 min and by two washes in cold PBS. Afterward, samples were lysed and sonicated with M220-Focused Ultra Sonicator (Covaris) to obtain fragments of 250–500 bp. Samples were processed according to Blueprint Histone ChIP-Seq protocol (https://www.blueprint-epigenome.eu/). The following antibodies were used for immunoprecipitation: 2.5 μg of anti-H3K27me3 (07449) and 2.5 μg of anti-H3K27ac (ab4729). As experimental control we used input sonicated chromatin (not immunoprecipitated) in all experimental conditions. For the analysis, reads were checked for quality using FastQC (0.11.5) and then trimmed using trim galore (v.0.6.6) to remove the sequencing adapters. Reads were aligned to the mouse reference genome GRCm38 using Bowtie v2.3.2 with ‘–very-sensitive’ parameters (36). Aligned reads were then filtered based on ENCODE standards and removed those mapping to the blacklist and duplicates using samtools (v.1.9) and sambamba (v.0.7.0). Peaks were called using MACS2 v2.2.7.1 (37) with default parameters, providing an input sample to avoid false positives. Background correction was applied by first defining a set of non-redundant enriched regions for all samples by taking the union of all peaks from both replicates of all samples. Bigwig files were generated using bamCoverage v3.2.1 from deeptools (38). Genomic peak annotation was performed with HOMER software (v4.11). Intensity plots were performed using computeMatrix in a window of ±1 kb center in the TSS from deeptools (38). Data from H3K27ac and H3K27me3 ChIP experiments are available under accession code: GSE204673.

### ATAC-seq experiment and analysis

50 000 purified pro-B and pre-B cells from the bone marrow of *Hdac7*^+/−^ and *Hdac7*^fl/−^ mice were isolated and freshly lysed using cold lysis buffer (10 mM Tris−HCl, pH 7.4, 10 mM NaCl, 3 mM MgCl_2_ and 0.1% IGEPAL CA-630). Immediately after lysis, nuclei were spun at 500 g for 10 min using a refrigerated centrifuge, and pellet was resuspended in the transposase reaction mix (12.5 μl 2 × TD buffer, 2 μl transposase (Illumina) and 5.5 μl nuclease-free water). The transposition reaction was carried out for 1 h at 37°C, followed by addition of clean up buffer (900 mM NaCl, 300 mM EDTA, 2 μl 5%SDS, 20 ng Proteinase K) and incubation for 30 min at 40°C. Tagmented DNA was isolated using 2× SPRI beads from Beckman–Coulter. Following purification, we amplified library fragments using 1× NEBnext PCR master mix and 1.25 μM of Nextera PCR primers as described elsewhere (39). Sequence reads quality was assessed with MultiQC v1.12 (40), and adapter content (if any) was trimmed using Trimmomatic v0.39 (41). Paired-end reads were aligned to GRCm38 using Bowtie2 v2.2.3 with a maximum insert size of 2000 (-X 2000) (36). These parameters ensured that fragments up to 2 kb were allowed to align and that only unique aligning reads were collected (-m1). For all data files, duplicates and mtDNA were removed using picard (http://picard.sourceforge.net) and sambamba (v.0.7.0), respectively. For peak-calling, MACS2 v2.2.7.1 (37) tool was used with default parameters. Background correction was applied by first defining a set of non-redundant enriched regions for all samples by taking the union of all peak summits from both replicates of all samples. We then quantified the signal at all summits in each sample by counting the number of fragments (using the R bioconductor package csaw, v. 1.0.7) (42). The resulting counts matrix file was analyzed for differential peaks with DESeq2 (43). Genomic peak annotation was performed with HOMER software (v4.11). Intensity plots were performed using computeMatrix in a window of ±1 kb center in the TSS from deepTools (38). Data from ATAC-seq are available under accession code GSE204672.

### Quantification of global 5-hydroxymethylation levels

To quantify 5-hmC, a Quest 5hmC DNA ELISA kit (Zymo Research) was used according to the manufacturer's protocol. First, genomic DNA from sorted cells was extracted using Quick-DNA Miniprep Plus kit (D4068, Zymo Reseach). Next, the bottom of the provided well was coated with anti-5-hmC polyclonal antibody (pAb) for 1 h at 37°C in the dark. Wells were then blocked and 100 ng of denatured genomic DNA was added for 30 min at 37°C in the dark. After corresponding washes, anti-DNA HRP antibody was applied to wells for 30 min at 37°C in the dark. After corresponding washes, HRP developer (3,3’,5,5’-tetramethylbenzidine (TMB) (Sigma-Aldrich)) was added to detect the DNA bound to the anti-5-hmC pAb for 20–30 min at room temperature in the dark. Afterward, the color reaction was stopped by the addition of sulfuric acid and the resulting color was analyzed at 450 nm by using a Glomax microplate reader (Promega). The percentage of 5-hmC DNA was estimated from linear regression.

### hMeDIP-qPCR experiments

Genomic DNA was purified by using the same kit as in ELISA assay. 1 μg of genomic DNA from wild-type and HDAC7-deficient sorted pro-B cells was sonicated with M220-Focused Ultra Sonicator (Covaris) to obtain fragments of 300–400 bp. Fragmented DNA were incubated with 2 μg anti-5hmC (Active Motif, 39769) and 20 μl of Dynabeads G (Life Technologies) for 16 h at 4°C, and 10% of DNA was kept to be used as input. After incubation, Dynabeads were washed 3 times with IP buffer (10 mM Na-Phosphate pH 7, 0.14 M NaCl, 0.05% Triton X-100) and then were resuspended in Proteinase K digestion buffer (50 mM Tris pH8, 10 mM EDTA, 0.5% SDS) for 30 min at 55°C. DNA from immunocomplexes was purified with the QIAquick MinElute kit (Qiagen). Real-time quantitative PCR was performed and the results analyzed. Data are presented as the ratio of the enrichment of 5-hmC relative to the input control.

### hMeDIP sequencing experiments and analysis

Purified genomic DNA (1 μg) from wild-type and HDAC7-deficient pro-B cells was sonicated to obtain fragments of 300–400 bp. Adaptor ligations were performed and libraries constructed by qGenomics Laboratories (Barcelona, Spain). 2 μg of anti-5hmC (Active Motif, 39769) were incubated with 20 μl of Dynabeads G (Life Technologies) for 2 h at 4°C. Fragmented DNA was incubated with Dynabeads and antibody for 16 h at 4°C, and 10% of DNA was kept to be used as input. DNA was purified as described in hMeDIP qPCR assay. Amplified libraries were constructed and sequenced at qGenomics Laboratories (Barcelona, Spain). Fastq data were obtained with Trim Galore-0.4.2 and Cutadapt-1.6. Reads were mapped with bwa-0.7.12. Sorting Sam to Bam was carried out with Picard-2.8.0 SortSam and duplicates were removed with Picard-2.8.0 MarkDuplicates. Bigwig files were made with deeptools and normalized based on RPKM. Peak calling was performed using MACS2 bdgpeakcall option (-c 250 -l 10 -g 10). To avoid false positives, peaks that intersect with peaks in the corresponding input samples were removed. DESEQ analysis (DESeq2 v1.20.0) was then used to define peaks and perform quantitative analyses. The Diffbind-2.6.6 R package was used for differential binding analysis. Differential enrichment was defined by a threshold value of *P* = 0.005 and a >1-fold difference in KO relative to WT samples. Motif enrichment was analyzed and peak depth quantified with HOMER software (v4.10). Data from hMeDIP-seq is available under accession code GSE135263.

### Co-immunoprecipitation (Co-IP)

Co-IP assays were performed using CD19^+^ cells from control and HDAC7 deficient mice. Cell extracts were prepared in lysis buffer [50 mM Tris–HCl, pH 7.5, 1 mM EDTA, 150 mM NaCl, 1% Triton-X-100, protease inhibitor cocktail (complete™, Merck)] with corresponding units of Benzonase (Sigma) and incubated at 4°C for 6 h. 50 μl of supernatant was saved as input and diluted with 2× Laemmli sample buffer (4% SDS, 20% glycerol, 120 mM Tris–HCl, pH 6.8). Supernatant was first incubated with PureProteome™ Protein A/G agarose suspension (Merck Millipore) for 1 h to remove background signal. Samples were then incubated overnight at 4°C with corresponding antibodies against TET2 (ab124297, Abcam) and rabbit (12-371, Merck Millipore) IgGs (negative control) and A agarose beads. After that, beads were washed three times with lysis buffer. For sample elution, 100 μl of 1× Laemmli sample buffer was added to the beads. Samples and inputs were denatured at 95°C in the presence of 1% β-mercaptoethanol.

### Expression profiling of microRNAs

We used miRCURY LNA™ Universal RT microRNA PCR System (Exiqon) to determine miRNA expression profiles. The miRNA annotation of mirBase version 20.0 was used. Single-stranded cDNA was synthesized by reverse transcription of 8 μl of RNA, using the universal cDNA Synthesis Kit II (Exiqon). Diluted cDNA was mixed with ExiLENT SYBR^®^ Green master mix (Exiqon), and quantitative PCR was performed using the Roche LightCycler^®^ 480 RealTime PCR system (Roche). Primers design for validations of miRNA’s differential expression was performed with miRprimer software (https://sourceforge.net/projects/mirprimer/).

### Immgen data analysis

Immgen is a public resource that is the result of a collaborative group of immunology and computational biology laboratories that share knowledge and expertise to perform a broad and deep dissection of the genome's activity and its regulation in the immune system. This public resource is broadly used by many immunology laboratories to interrogate biological questions of a gene of interest or more broad mechanistic insights within the hematopoietic system.

Gene Skyline tool: expression data from microarrays collected from several participating laboratories is normalized with Robust Multiarray Average (RMA) algorithm (44,45). RMA is a three-step algorithm, which removes background noise (normally distributed), performs quantile normalization and log2 transformation. Raw reads from microarray are normalized by median of ratios with DeSeq2 package from Bioconductor. Spike-in data is also used to evaluate the RMA method in order to address questions about the correctness of microarray data. Immgen developers ensure that the comparison of expression levels of one gene among different cell types is sensitive and quantitative.

Modules and regulators tool: this tool uses an algorithm called Ontogenet in order to outline the regulatory networks that drive the hematopoietic cell differentiation (see detailed explanation in (46). Briefly, it classifies and clusters genes in groups called ‘modules’ based on expression levels of genes among all cell types from all lineages and developmental stages from the hematopoietic system given by several microarray analyses. Each module is called ‘coarse module’, which can be further divided into ‘fine modules’. The algorithm assumes that genes included in one module are regulated in the same way. For these modules, Immgen create automatically an expression matrix represented as a heatmap, showing potential regulators of the module and their expression pattern among blood cell types. Immgen also provides a table of expression level and regulatory activity (weight), classifying each regulator into positive and negative.

My GeneSet tool: this browser allows you to examine the pattern of expression of a selected set of genes over some or all of the ImmGen expression data (obtained from either RNA-seq or microarray experiments).

### Statistical analysis

Data were analyzed by Student's two-tailed unpaired *t*-test and Mann–Whitney test using GraphPad Prism (v7). *P-*values lower than 0.05 were considered statistically significant. Statistical methods for analysis of genome wide datasets involving hMeDIP-seq, RNA-seq, ChIP-seq and ATAC-seq are explained in detail under the respective section.

## RESULTS

### HDAC7 regulates chromatin condensation and 5-hmC levels in pro-B cells

**Figure 1.**
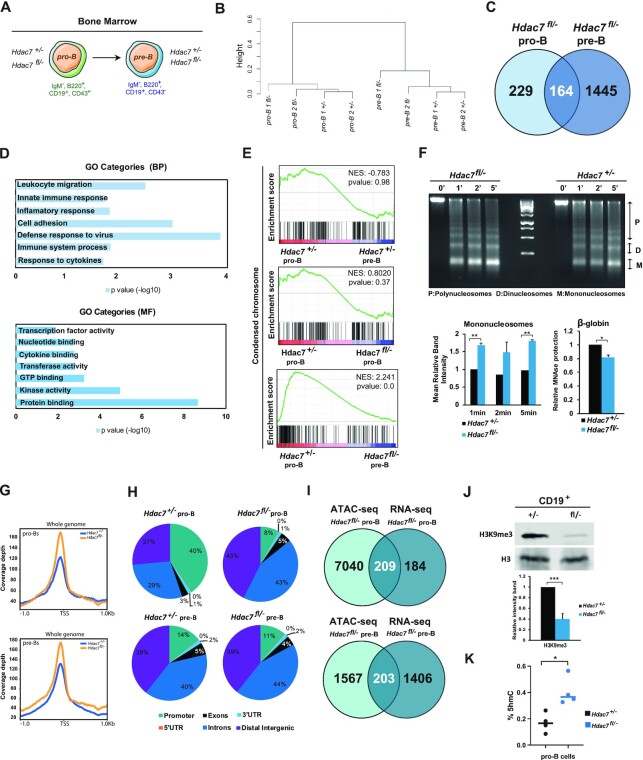
HDAC7 deficiency results in global chromatin de-condensation and increase 5-hmC levels. (**A**) Experimental design for RNA-seq experiments, showing the type of cells used. B cell progenitors (pro-B); B cell precursors (pre-B). (**B**) Cluster dendogram obtained from RNA-seq gene expression profiles for each replicate and cell type. (**C**) Venn Diagram comparing up-regulated genes in *Hdac7^fl/–^* pro-B cells respect to control pro-B cells (left) and in *Hdac7^fl/–^* pre-B cells respect to control pro-B cells (right). (**D**) Gene ontology (GO) enrichment analysis of up-regulated genes in HDAC7 deficient pro-B and pre-B cells respect to control pro-B cells. BP refers to Biological Processes and MF corresponds to Molecular Functions. (**E**) GSEA analysis comparing wild-type pre-B and HDAC7-deficient pro-B and pre-B cells respect to wild-type pro-B cells in expression profiles related to condensed chromosome signatures. (**F**) Chromatin accessibility assay in control and HDAC7-deficient B cells. Isolated nuclei from these cells were digested with 5 units of micrococcal nuclease (MNase) for 0, 1, 2 or 5 min. Different nucleosomal fractions (mono, di and polynucleosomes) were separated by gel electrophoresis (upper panel). Band density quantification from chromatin accessibility assay using ImageJ software. Results are expressed as the mean ± SEM of three independent experiments. **P < 0.01 by unpaired *t*-test (left lower panel). qPCR showing protection of MNase-digested DNA after 5 min incubation in control or HDAC7-deficient B cells (right lower panel). Data represent mean ± SEM of three independent experiments per condition after normalization to *Actb* gene expression. *P < 0.05 by unpaired T-test. (**G**) ATAC-seq coverage depth (per base pair per peak per 10 million mapped reads) of peaks located in the TSS (−1 kb to +1 kb) in wild-type and HDAC7-deficient pro-B (upper panel) and pre-B cells (lower panel). (**H**) Genomic distribution of chromatin accessible peaks in *Hdac7^+/–^* and *Hdac7^fl/–^* pro-B and pre-B cells determined by ATAC-seq experiments. (**I**) Venn Diagram comparing differentially enriched peaks in ATAC-seq in *Hdac7^fl/-^* pro-B cells and up-regulated genes in RNA-seq in *Hdac7^fl/-^* pro-B cells (upper panel) and pre-B cells (lower panel) compared to control cells. (**J**) H3K9me3 global levels decrease upon HDAC7 deficiency, analyzed by Western blot representative assay (upper panel). Total H3 levels were used as a control. Band quantification of the Western blot experiment was performed using ImageJ program. Results are expressed as the mean ± SEM of three independent experiments. ***P < 0.001 by unpaired *t*-test (lower panel). (**K**) ELISA assay showing global levels of DNA 5-hmC in pro-B cells from wild-type and HDAC7-deficient mice. Each dot represents one animal (*n* = 4, test); the line represents the mean. Data is represented as mean ± SEM of four independent experiments. *P < 0.05, Mann–Whitney test.

We previously identified HDAC7 as a critical transcriptional repressor of lineage-inappropriate genes that ensures correct early B cell development. However, its exact molecular mechanisms remained to be addressed (26,30). To unveil the mechanisms and functions that HDAC7 exerts during early B lymphocyte development along the different cell stages of differentiation, we performed RNA-sequencing (RNA-seq) experiments on pro-B and pre-B cells from HDAC7 conditional knockout mice (*Hdac7*^fl/–^; hereafter, HDAC7-deficient) and their control littermates (*Hdac7*^+/–^; hereafter, wild-type) (Figure 1A; see also gating strategy, Supplementary Figure S1A). First, we performed an unsupervised cluster analysis of the samples based on their gene expression profiles. Notably, pro-B and pre-B wild-type cell populations were grouped into two different clusters, confirming that early B cell differentiation steps were affected by gene transcription changes, a hallmark of early B lymphocyte development. In contrast, HDAC7-deficient pro-B and pre-B cells were more similar and grouped closer, indicating that the B cell developmental block observed upon HDAC7 deficiency is a consequence of altered gene transcription programs (Figure 1B). Based on RNA-seq analyses, a comparison of the differentially expressed genes between the cell populations showed that 1992 and 2140 genes were up-regulated and down-regulated, respectively, during the cellular transition from pro-B to pre-B wild-type cells (Supplementary Figure S1B). The lack of HDAC7 from the pro-B or pre-B cells resulted in a downregulation of 272 and 1576 genes, respectively, as compared to the wild-type pro-B cells (Supplementary Figure S1B). Among the downregulated genes, we observed a dramatic disruption in heavy chain immunoglobulin (Igh) production (Supplementary Figure S1C), which is a critical process in early B cell development; this demonstrated that HDAC7 deficiency would drastically affect the immune response (Supplementary Figure S1D).

HDAC7 is a transcriptional repressor. Accordingly, 393 and 1609 genes in pro-B and pre-B cells, respectively, were upregulated in the HDAC7-deficient cells as compared to pro-B wild-type cells (Figure 1C). Gene Ontology (GO) analysis of up-regulated genes at both cell stages showed that they were related, on one hand, to cell proliferation, survival and immune processes (Supplementary Figure S1E) and, on the other hand, to enhanced protein binding (Supplementary Figure S1F). To specifically analyze the gene expression changes exclusively depending on HDAC7, and omit differences due to different cell stages, we overlapped upregulated genes from pro-B and pre-B cells. GO analysis revealed that overlapped upregulated genes upon HDAC7 deficiency were associated with an altered immune response (Figure 1D, upper panel), with a clear tendency for increased protein binding and transcription factor (TF) activity, which may correlate with a more open chromatin state (Figure 1D, lower panel). Gene set enrichment analysis (GSEA) showed that the gene signatures in HDAC7-deficient pro-B or pre-B cells were associated to a deficiency in chromosome condensation, suggesting a potential loss of the heterochromatin state in the absence of HDAC7 (Figure 1E). This de-condensation was independent of the cell stage, as there was no difference in these gene signatures between control pro-B and pre-B cells, when separately analyzed for each genotype (Figure 1E). These results indicate that HDAC7 deficiency may result in chromatin decompaction. Accordingly, HDAC7-deficient B cells presented increased chromatin accessibility, as genomic DNA from these cells was more sensitive to micrococcal nuclease (MNase) digestion than that from control B cells (Figure 1F, upper and lower left panels). These results were reinforced by qPCR showing protection of MNase-digested DNA at β-globin site (Figure 1F, lower right panel). To definitively prove the potential role of HDAC7 in maintaining proper chromatin status in pro-B and pre-B cells, we performed transposase-accessible chromatin assay coupled with next generation sequencing (ATAC-seq). We found that the global chromatin accessibility intensity was higher in both HDAC7-deficient pro-B and pre-B cells compared to control cells (Figure 1G). Consistent with its role as a transcriptional repressor, we observed ∼13 000 more accessible peaks in HDAC7-deficient pro-B cells. Next, we determined ATAC-seq differential regions between HDAC7-deficient and wild-type pro-B (Figure 1H, upper panel) and pre-B cells (Figure 1H, lower panel). We found that the genomic distribution of the accessible sites differed significantly in the absence of HDAC7. In particular, we observed a reduction of peaks located at promoters and, more notably in pro-B cells, a dramatic increase of accessible peaks at distal intergenic regions (Figure 1H). To interrogate a potential functional link between the altered chromatin landscape and transcriptional programming in the absence of HDAC7, we integrated the data obtained in ATAC-seq and RNA-seq experiments. We compared the open chromatin regions in HDAC7-deficient pro-B and pre-B cells versus upregulated genes in the absence of HDAC7 (Figure 1I, upper panel). Half of the differentially upregulated genes (209 genes) in pro-B cells also presented a more open chromatin state in HDAC7-deficient cells, whereas 203 genes among the pre-B cells upregulated genes set showed a more accessible chromatin (Figure 1I, lower panel). Additionally, and given that chromatin condensation is associated to heterochromatin state, we next isolated CD19^+^ B cells from HDAC7-deficient or wild-type mice and assessed the levels of H3K9me3, a well-known epigenetic mark involved in the establishment of heterochromatin, genome stability, and cell identity maintenance (47). We found that HDAC7-deficient B cells showed a significant and dramatic decrease in H3K9me3 as compared to their wild-type counterparts (Figure 1J). Increases in 5-hmC have been associated with chromatin decompaction and, consequently, with alterations in hematopoietic cell differentiation and the maintenance of cell identity (48). Importantly, 5-hmC and H3K9me3 have an opposite genomic localization pattern, with TET proteins recruited mostly to euchromatin regions (49,50). We next carried out ELISA assays to determine the global 5-hmC levels; which revealed that HDAC7 loss in pro-B cells led to a significant increase in the global levels of 5-hmC (Figure 1K). Thus, altogether our data indicated that HDAC7 controls chromatin compaction and DNA 5-hmC levels at early stages of B cell development.

### HDAC7 regulates TET2 expression in pro-B and pre-B cells

**Figure 2.**
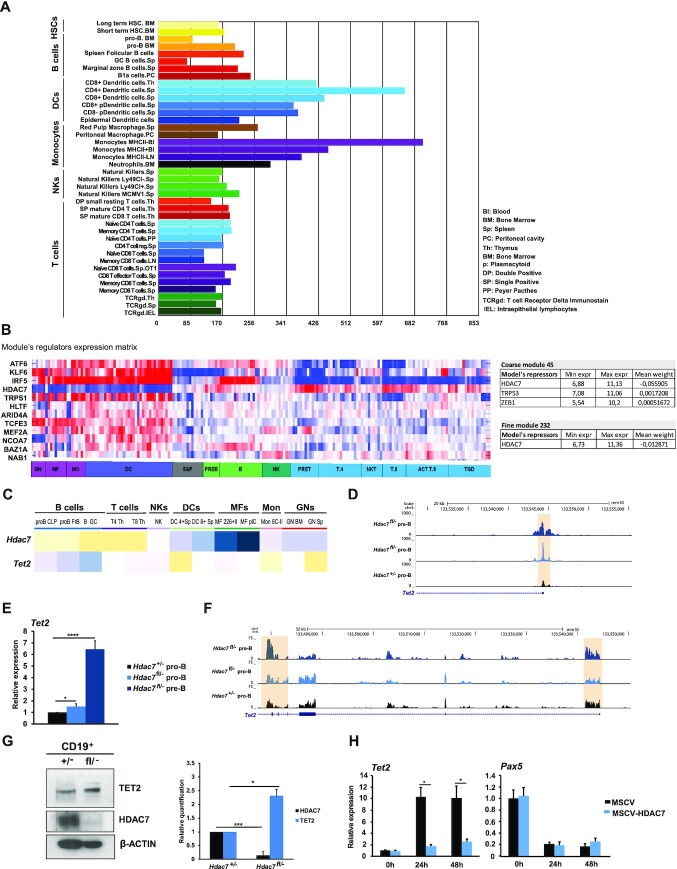
(A) HDAC7 controls DNA 5-hmC through *Tet2* regulation in pro-B and pre-B cells. Gene skyline showing the *Tet2* gene expression profile along different murine hematopoietic cell lineages (obtained from ‘gene skyline’ tool of Immgen database). (**B**) Heatmap showing the expression of potential *Tet2* gene regulators in hematopoietic cell subsets (left panel). Tables showing mean weight (enzymatic activity) of potential negative regulators of *Tet2* gene in hematopoietic cells. *Tet2* gene is included in two modules or cluster of genes: coarse module includes 315 genes and three negative regulators and fine module contains 35 genes and one negative regulator (right panel). Data were extracted from Immgen public database using *Modules and Regulators* tool. (**C**) Heatmap showing the inverse expression of *Hdac7* and *Tet2* in the indicated cell subsets. Data was extracted from Immgen public database using *My Gene Set* tool. (**D**) Genome browser snapshot of the *Tet2* gene showing signal for RNA-seq in wild-type and HDAC7-deficient pro-B and pre-B cells. Orange shadow indicates *Tet2* promoter. (**E**) Analysis by RT-qPCR of the *Tet2* mRNA levels in wild-type and HDAC7-deficient pro-B and pre-B cells. (**F**) Genome browser snapshot of the *Tet2* gene showing signal for ATAC-seq in *Hdac7^+/^^−^* pro-B cells, *Hdac7^fl/^^−^* pro-B and pre-B cells. Orange shadows indicate *Tet2* promoter and additional exonic regions. (**G**) TET2 upregulation in *Hdac7^fl/–^* CD19^+^ B cells compared to *Hdac7^+/–^* B cells analyzed by Western Blot assay (left panel). Quantification of two Western Blot experiments was performed by using ImageJ program (right panel). (**H**) RT-qPCR experiments for gene expression changes for *Tet2* and *Pax5* in the absence or presence of HDAC7 during the conversion of pre-B cells into macrophage-like cells. 24 and 48 h time points indicate time since transdifferentiation induction with β-estradiol. Data from E and H is represented as mean ± SEM of four independent experiments. *P < 0.05, ****P < 0.0001, unpaired *t*-test. Data from G is represented as mean ± SEM of two independent experiments. * *P* < 0.05, unpaired *t*-test.

Our results suggested that an essential function of HDAC7 during early B cell development could be the regulation of TET proteins expression, which are responsible for incorporating 5-hmC into DNA. Among the different family members, TET2 appears to play crucial roles during hematopoiesis and is highly expressed in myeloid cells (20–22). Therefore, we hypothesized that HDAC7 may be responsible for tightly regulating and fine-tuning the physiological levels of TET2 in pro-B and pre-B cells, thereby ensuring proper cell differentiation and identity. We directly addressed our hypothesis firstly by analyzing whether TET2 is expressed at different levels in the lymphoid and myeloid compartments within the hematopoietic system. For this purpose, we examined transcriptomic data from the Immunological Genome Project Database (Immgen) (http://www.immgen.org/). Indeed, TET2 was expressed at much higher levels in myeloid cells than in lymphoid populations (Figure 2A). Next, taking advantage of the ‘modules and regulators’ interactive tool in the Immgen database, we searched for putative positive and negative TET2 regulators. This tool uses all expression data of hematopoietic cell populations deposited by several groups and enables the search for putative positive and negative regulators of the gene of interest. Strikingly, HDAC7 appeared to be the only negative regulator or transcriptional repressor controlling TET2 expression within the hematopoietic system (Figure 2B). Further analysis of data from Immgen database to compare the expression pattern between HDAC7 and TET enzymes coding genes in lymphoid and myeloid populations revealed an inverse correlation with gene expression between HDAC7 and TET2 (Figure 2C), indicating a fully opposite expression pattern of both genes throughout hematopoiesis. In contrast, TET1 had an expression pattern similar to that of HDAC7, while TET3 displayed uniform expression levels throughout all tested cell populations (Supplementary Figure S2A and B). These data suggested that HDAC7 was responsible for controlling TET2 levels in lymphoid cells. We next examined data from RNA-seq in Figure 1B–E, as well as from our published microarray (26). We found that *Tet2* was upregulated in HDAC7-deficient pro-B and pre-B cells with respect to control pro-B cells (Figure 2D and E) reaching similar levels to that of macrophages (Supplementary Figure S2C), whereas the expression of the essential B cell genes *E2a* and *Pax5* remained unaltered (Supplementary Figure S2D and E). RT-qPCR assays revealed that the absence of HDAC7 from pro-B and pre-B cells did not alter the expression levels of the other class IIa HDACs family members (HDAC4, 5 and 9) indicating the specific requirement of HDAC7 during early B cell development (Supplementary Figure S2F). According to mRNA levels, analysis of our ATAC-seq revealed a more open chromatin at *Tet2* gene loci in HDAC7-deficient pro-B cells and pre-B cells (Figure 2F). Western blot experiments confirmed that the absence of HDAC7 from B cells corresponded with significantly increased TET2 protein levels (Figure 2G). Therefore, different physiological levels of TET2 protein may have an effect and specific function in hematopoietic cell populations. Next, using a transdifferentiation system developed by Graf laboratory (24), we found that HDAC7 exogenous expression blocked the upregulation of *Tet2* during pre-B cells conversion into macrophages (Figure 2H). Forced expression of HDAC7 did not interfere with the downregulation of *Pax5* during cellular conversion (Figure 2H), confirming that its expression is totally independent of that of HDAC7. Together, these findings indicated that HDAC7 may govern early B cell development in the bone marrow through the control of the physiological levels of TET2.

### *Tet2* is a direct HDAC7 target gene in pro-B cells

**Figure 3.**
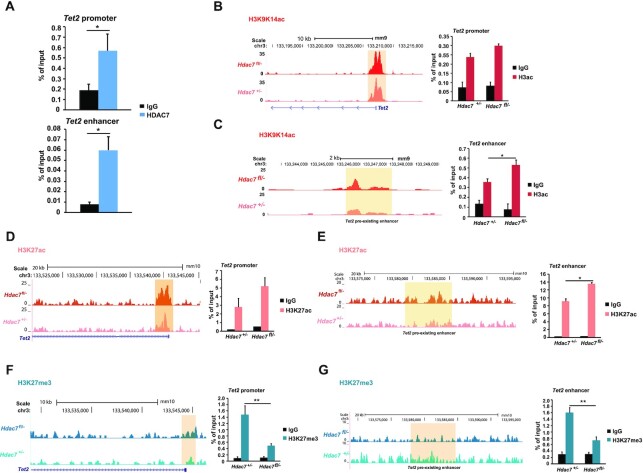
*Tet2* is a direct HDAC7 target gene in pro-B and pre-B cells. (**A**) Chromatin immunoprecipitation (ChIP) experiments showing the recruitment of HDAC7 to the *Tet2* promoter or the *Tet2* enhancer, quantified as % of input. (**B**) Genome browser snapshot of H3K9/K14ac ChIP-seq (left panel) showing differential enrichment levels at the promoter region of *Tet2* gene for HDAC7-deficient pro-B and control cells. ChIP-qPCR assay validating ChIP-seq data after immunoprecipitation with anti-H3K9/14ac antibody is shown in right panel, quantified as % of input. (**C**) As in (B), but for a *Tet2* pre-existing enhancer region. (**D**, **E**) As in (B, C), but for H3K27ac ChIP-seq enrichment and after immunoprecipitation with H3K27ac antibody. (**F**, **G**) As in (B, C), but for H3K27me3 ChIP-seq enrichment and after immunoprecipitation with H3K27me3 antibody. All data are represented as mean ± SEM of *n* = 3. *P < 0.05, **P < 0.01, unpaired *t*-test.

We next performed chromatin immunoprecipitation (ChIP) assays to test whether *Tet2* is a direct HDAC7 target gene in B cells; we found that HDAC7 was recruited to the promoter of the *Tet2* gene, which contains MEF2 binding sites in pro-B cells (Figure 3A, upper panel). This was consistent with the previously demonstrated requirement of HDAC7 to interact with MEF2C to repress its target genes in pro-B cells (26), and with *Tet2* being a MEF2C direct target in pro-B cells (6). We also demonstrated HDAC7 recruitment at a previously described *Tet2* enhancer (24) (Figure 3A, lower panel). We previously reported by ChIP-seq experiments that HDAC7 loss in pro-B cells causes an increase of H3K9/K14ac at the promoter and enhancers of its target genes (26). ChIP-seq data examination and ChIP-qPCR revealed an increase of H3K9/K14ac at both promoter and enhancer regions of *Tet2* in the absence of HDAC7 from pro-B cells (Figure 3B and C). As expected, the absence of HDAC7 from pro-B cells had no effect on H3K9/K14ac enrichment at *Pax5* gene (Supplementary Figure S3A). Next, integration of ATAC-seq and H3K9/K14ac ChIP-seq data from HDAC7-deficient pro-B cells revealed an overlapping of 4487 enriched regions, remarkably including *Tet2* (Supplementary Figure S3B). To further gain insight into the mechanisms altered by HDAC7 deficiency, we performed ChIP-seq of H3K27ac and H3K27me3 histone marks in wild-type and HDAC7-deficient pro-B cells. The analysis revealed an increased global intensity of the activating histone mark H3K27ac in the HDAC7-deficient pro-B cells (Supplementary Figure S3C), as well as at both specific promoter and enhancer regions of *Tet2* gene (Figure 3D, E). In addition, the absence of HDAC7 from pro-B cells resulted in a decrease of global H3K27ac mark in promoter regions and an increase in distal intergenic regions (Supplementary Figure S3D). Increased number of H3K27ac peaks in distal regions upon HDAC7 deficiency agrees with previous results of more open chromatin regions under the same conditions, reinforcing the repressive role of HDAC7. The integration of ATAC-seq and H3K27ac-seq data revealed 3113 common peaks (representing ∼40% of the enriched regions in both assays) in the absence of HDAC7, in which *Tet2* was also included (Supplementary Figure S3E). In parallel, H3K27me3 ChIP-seq data unveiled similar coverage intensity between wild-type and HDAC7 deficient pro-B cells (Supplementary Figure S3F). The genomic distribution of H3K27me3 remained also unaltered in the absence of HDAC7 (Supplementary Figure S3G). In the case of H3K27me3 enrichment in HDAC7-deficient pro-B cells, we performed the integrative analysis comparing with ATAC-seq data from wild-type cells and observed an overlap of ∼25% of enriched peaks (Supplementary Figure S3H). ChIP-qPCR demonstrated a significant decrease of H3K27me3 at *Tet2* gene loci in HDAC7-deficient pro-B cells (Figure 3F, G). Finally, we aimed to determine if the global decrease in H3K9me3 in the absence of HDAC7 shown in Figure 1J could be observed at the *Tet2* gene loci. Indeed, ChIP-qPCR showed that HDAC7 deficiency in pro-B cells led to lower enrichment levels of the repressive histone mark H3K9me3, at both the promoter and enhancer of *Tet2* gene (Supplementary Figure S3I). Collectively, our findings demonstrated that HDAC7 controls the proper deposition of epigenetic marks and that *Tet2* is a direct HDAC7-target gene in pro-B cells.

### HDAC7 deficiency alters 5-hmC at specific loci

**Figure 4.**
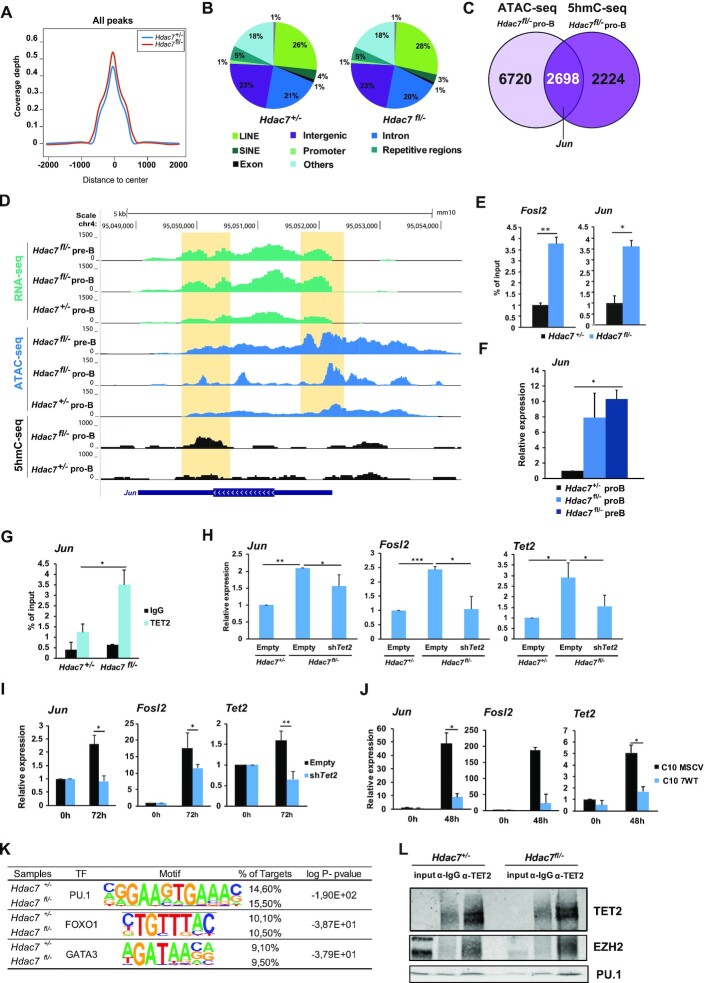
HDAC7 deficiency results in increased recruitment of TET2 and altered 5-hmC enrichment at B cell lineage inappropriate genes. (**A**) 5-hmC coverage depth (per base pair per peak per 10 million mapped reads) of 5-hmC peaks (−2 kb to +2 kb) in control and HDAC7-deficient pro-B cells. (**B**) Genomic distribution of 5-hmC enrichment in *Hdac7^+/–^* and *Hdac7^fl/–^* pro-B cells. (**C**) Venn Diagram comparing enriched peaks associated genes in ATAC-seq from *Hdac7^fl/−^* pro-B cells and 5hmC enriched genes in hMeDIP-seq in *Hdac7^fl/−^* pro-B cells compared to control cells. (**D**) Genome browser snapshot of the *Jun* gene showing signal for 5-hmC from hMeDIP-seq, ATAC-seq and RNA-seq data in wild-type or HDAC7-deficient pro-B and pre-B cells. The peak locations are indicated in the orange-shaded rectangles. Orange shadows indicate *Jun* gene regions with differential peaks. (**E**) 5hmC-qPCR experiments showing the enrichment of 5-hmC in wild-type or HDAC7-deficient pro-B cells in the *Fosl2* and *Jun* genes. (**F**) Analysis by RT-qPCR of *Jun* mRNA levels in control or HDAC7-deficient pro-B cells and pre-B cells. (**G**) Analysis of TET2 recruitment at the *Jun* gene by ChIP-qPCR in control and HDAC7-deficient pro-B cells. (**H**) *Ex vivo* shCtrl and shTet2 retroviral infection in B cells from HDAC7-deficient mice. As control, wild-type B cells were transduced with shCtrl vector. Cells were infected with sh*Tet2* and expression levels of target genes were analyzed by RT-qPCR 72h after retroviral infection. (**I**) *In vitro* shTet2 infection of C11 pre-B cells. Cells were infected with shCtrl or shTet2 and then treated with β-estradiol to induce trans-differentiation to macrophages. Expression levels of target genes were analyzed after 72 h of transdifferentiation (**J**) C10 pre-B cells expressing empty plasmid (MSCV) and HDAC7-expressing plasmid (7WT) were treated with β-estradiol to induce transdifferentiation to macrophages. Expression levels of target genes were analyzed after 48 h of transdifferentiation. (**K**) The most relevant TF binding motifs in wild-type or HDAC7-deficient pro-B cells, based on their enrichment in 5-hmC using the HOMER database of known motifs. (**L**) Co-immunoprecipitation assays performed in *Hdac7^+/–^* and *Hdac7^fl/–^* CD19^+^ B cells. Protein extracts were immunoprecipitated using an anti-TET2 antibody, using IgG as a negative control and total protein extract as input. Data in (E, F) are represented as mean ± SEM of *n* = 4. Data in (G–J) are represented as mean ± SEM of *n* = 3. *P < 0.05, **P < 0.01, ***P < 0.001 unpaired *t* test.

We next performed a 5-hmC DNA immunoprecipitation (hMeDIP) followed by next-generation sequencing (hMeDIP-seq) in pro-B cells purified from *Hdac7^fl/–^* mice and their *Hdac7*^+/–^ control littermates. While the total frequencies of 5-hmC peaks were similar, the global 5-hmC coverage was higher in HDAC7-deficient pro-B cells than in wild-type pro-B cells, in concordance with the results from Figure 1K (Figure 4A and Supplementary Figure S4A). The genomic distribution of the peaks did not differ significantly between the two genotypes, with most peaks located in intergenic and intronic regions as well as within LINE-1 elements (Figure 4B). Further, we found differential peaks in 5-hmC enrichment: HDAC7-deficient pro-B cells had an increase of ∼13 000, and a decrease in ∼15 800, 5-hmC peaks, as compared to wild-type B cells. Next, we performed an integrative analysis of the ATAC-seq and hMeDIP-seq data obtained. In particular, we overlapped the genes presenting a more open chromatin with the genes associated with higher 5-hmC enrichment in the HDAC7-deficient pro-B cells. We found that around 40% of genes with a more accessible chromatin harbored increased 5-hmC, indicating that HDAC7 may regulate a proper DNA compaction to avoid aberrant deposition of this epigenetic mark (Figure 4C). Focusing on alterations in potential lineage-inappropriate genes, we observed an increase in the enrichment of 5-hmC at the *Jun* gene in HDAC7-deficient pro-B cells as compared to wild-type cells, indicating that *Jun* may be aberrantly overexpressed in the absence of HDAC7 (Figure 4D). Indeed, RNA-seq data showed a higher expression of *Jun* in HDAC7-deficient pro-B and pre-B cells as compared to wild-type pro-B cells (Figure 4D). This correlated with a more open chromatin of the *Jun* gene in the absence of HDAC7 in both cell types (Figure 4D). Similar results were observed for the *Fosb* gene (Supplementary Figure S4B). The results from hMeDIP-seq and RNA-seq were confirmed by hMeDIP-qPCR and RT-qPCR experiments (Figure 4E, F, and Supplementary Figure S4C). Upregulation of additional lineage inappropriate genes, *Cd69* and *Itgb2*, in HDAC7-deficient pro-B and pre-B cells is shown in Supplementary Figure S4D. Importantly, we found increased TET2 recruitment to the *Jun* gene loci identified in the hMeDIP-seq analysis, as well as to further myeloid gene promoters (*Fosl2*, *Ahnak* and *Itgb2*), in HDAC7-deficient pro-B cells (Figure 4G and Supplementary Figure S4E).

To definitively prove the functionality of HDAC7–TET2 axis, we performed rescue analysis (gain and loss of function) using three different experimental approaches. A graphical scheme depicting the three experimental approaches is shown in Supplementary Figure S4F. First, we transduced purified B cells from bone marrow of wild-type and HDAC7-deficient mice with a retroviral vector for specific shRNA against *Tet2* (shTet2) and compared them to counterpart cells transduced with control retroviral vector (shCtrl). We found that knocking down *Tet2* prevented the upregulation of *Jun* and *Fosl2* in HDAC7 deficient B cells (Figure 4H). Therefore, as a second approach, we took advantage of the HAFTL murine pre-B cell line engineered to transdifferentiate into functional macrophages by addition of β-estradiol (C11 cells) and previously reported in (29). Similarly to the case of C10 cells, HDAC7 and TET2 become downregulated and upregulated during cellular conversion, respectively. C11 were transduced with shCtrl or shTet2 retroviral vectors and sorted GFP-positive cells were later induced to macrophage transdifferentiation by the addition of β-estradiol, in order to achieve double HDAC7 and TET2 deficiency (Figure 4I). RT-qPCR assays showed that *Jun* and *Fosl2* were upregulated after cellular conversion, concomitant to HDAC7 downregulation. Importantly, *Tet2* knockdown resulted in a significant block of both inappropriate genes induction (Figure 4I). And third, we determined the expression of *Jun* and *Fosl2* in C10 samples from Figure 2H similarly to the case of C11 cells, *Jun* and *Fosl2* were upregulated during the conversion of pre-B cells into macrophages. Importantly, HDAC7 exogenous expression blocked their increased expression (Figure 4J). These data demonstrate that the HDAC7–TET2 axis is involved in the proper control of the expression of lineage inappropriate genes in B cells.

Finally, we performed a motif enrichment analysis to determine whether HDAC7 deficiency produces an alteration in chromatin positioning that could lead to changes in TF occupancy. Although we found no differences associated with HDAC7, we did note enrichment of relevant TFs in the hematopoietic system, such as PU.1 (Figure 4K), which has been previously reported to interact with TET2 (51,52). The occupancy of PU.1 under both conditions is consistent with its relevance in both lymphoid and myeloid lineages. Accordingly, we corroborated that TET2 interacts with PU.1 in HDAC7-deficient B cells, as well as in wild-type B cells. As expected, EZH2 (a known TET2 partner) was also identified as an interactor (Figure 4L). Together, our results demonstrate an essential role of HDAC7 in silencing B cell-inappropriate genes by its regulation of TET2 expression and, consequently, of the DNA 5-hmC levels.

### HDAC7 directs 5-hmC and expression of specific miRNA in pro-B cells

**Figure 5.**
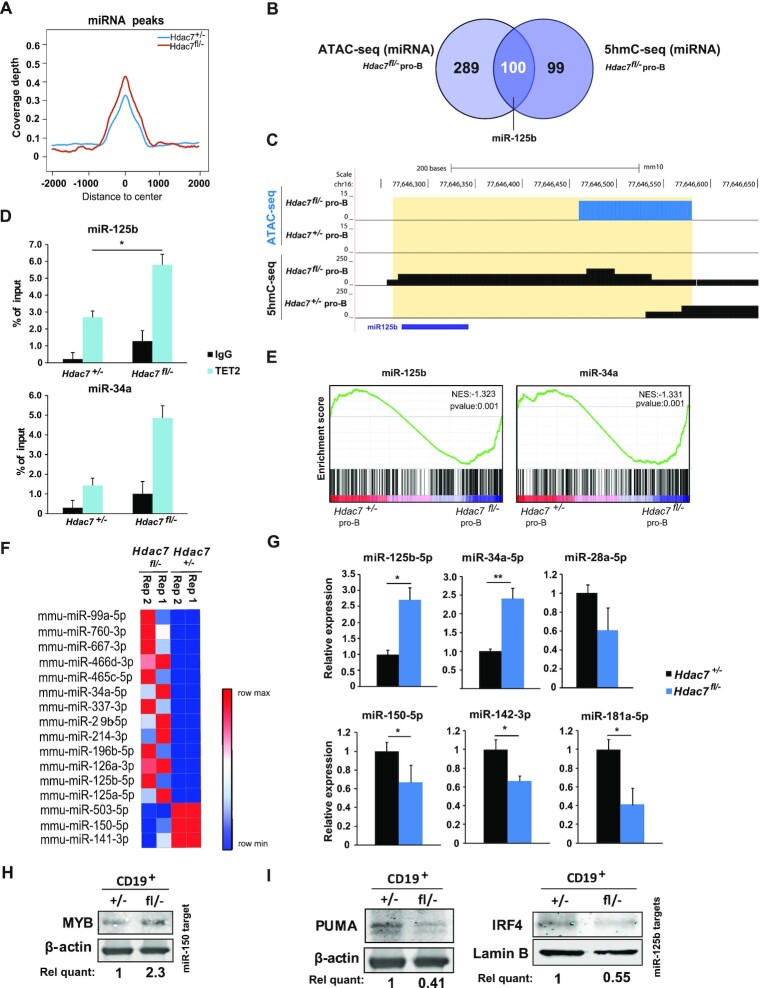
HDAC7 directs B cell–specific miRNA hydroxymethylation and expression patterns. (**A**) 5-hmC coverage depth (per base pair per peak per 10 million mapped reads) of 5hmC peaks located in microRNAs (−2 kb to +2 kb) from control or HDAC7-deficient pro-B cells. (**B**) Venn diagram comparing miRNA-related enriched peaks in ATAC-seq from *Hdac7^fl/−^* pro-B cells and miRNA-related peaks enriched in 5hmC-seq (or hMeDIP-seq) in *Hdac7^fl/−^* pro-B cells compared to control cells. (**C**) Example of 5-hmC and open chromatin enriched peaks at miR-125b from hMeDIP-seq and ATAC-seq experiments. (**D**) ChIP-qPCR analysis of TET2 recruitment to hydroxymethylated miRNAs in HDAC7-deficient pro-B cells. (**E**) GSEA analysis showing gene sets related to miR-34a and miR-125b functions, comparing HDAC7-deficient pro-B cells to control pro-B cells. miR-125B and miR-34a datasets were retrieved from www.gsea-msigdb.org. (**F**) Heat map of the differential expression of miRNAs for two HDAC7-deficient vs. wild-type replicates. Only miRNAs with a FC > 2 or FC < 0.5 2 from the miRCURY LNA™ Universal RT panel were selected. See also Supplementary Table S2. (**G**) RT-qPCR analysis of selected microRNAs from the miRCURY LNA™ Universal RT panel in HDAC7-deficient and control pro-B cells. The levels of U6 RNA were used for normalization. (**H**) Protein levels of miR-150 and miR-142 target MYB were assessed by western blot assays in control and HDAC7-deficient CD19^+^ cells. (**I**) Protein levels of miR-125b targets PUMA (left panel) and IRF4 (right panel) were assessed by western blot assays in control and HDAC7-deficient CD19^+^ cells. Using β-actin or Lamin-B as housekeepings, relative quantification of protein levels in G and H was performed with ImageJ software and is indicated below each panel. Data in (G) is represented as mean ± SEM of *n* = 3. *P < 0.05, **P < 0.01, unpaired *t* test.

Further examination of our hMeDIP-seq data revealed that the coverage depth of 5-hmC peaks at miRNAs in HDAC7-deficient pro-B cells was higher than in control pro-B cells (Figure 5A). Additionally, integrative analysis with our ATAC-seq obtained data demonstrated that more than 50% of miRNA-related peaks that are enriched in 5-hmC mark in the absence of HDAC7, are also located in open chromatin regions (Figure 5B). In fact, we found that several miRNAs involved in leukemia and lymphoma, as well as in myeloid differentiation, such as miR-125b and miR34a, were more enriched in 5-hmC and located in more open chromatin regions in pro-B cells from *Hdac7^fl/–^* mice (53) (see an example of miR125b in Figure 5C). Using ChIP-qPCR, we found that TET2 recruitment increased at both miR125b- and miR34a-associated loci, which correlated to the enhanced 5-hmC enrichment in HDAC7-deficient pro-B cells (Figure 5D). Additionally, GSEA analysis of our RNA-seq data confirmed that gene sets related to miR-34a and miR-125b functions were more expressed upon HDAC7 deficiency (Figure 5E). To examine a potential connection between changes in 5-hmC and chromatin condensation and HDAC7-mediated control of miRNA expression, we performed a miRNA profiling using a qPCR-based panel containing over 375 miRNAs (miRCURY LNA™ microRNA Array [Exiqon]) in wild-type or HDAC7-deficient pro-B cells (Figure 5F and Supplementary Table S2). We found 25 miRNAs whose levels of expression differed significantly between wild-type and HDAC7-deficient pro-B cells, which correlated with differential 5hmC enrichment and chromatin state. miRNAs that were up-regulated and 5hmC-enriched under HDAC7-deficient conditions included miR-125b-5p, miR-126, miR-29b, miR-34a and miR-99a (Figure 5F and Supplementary Figure S5A). On the contrary, B-cell related miRNAs that were down-regulated upon HDAC7 deficiency, such as miR-150a and miR-181a, also presented decreased 5hmC enrichment and more closed chromatin state (Figure 5F and Supplementary Figure S5B). The differential expression observed in HDAC7-deficient pro-B cells of several miRNAs involved in the hematopoietic system or related disorders were validated by RT-qPCR (Figure 5G). Thus, aberrant microRNAs (such as miR-125b and miR-34a) were upregulated, while B cell–specific miRNAs (such as miR-28a, miR150, miR-142 and miR181a) were downregulated in HDAC7-deficient pro-B cells. Finally, we tested whether targets of these miRNAs are altered as a consequence of HDAC7 deficiency. On one hand, we observed that protein levels of MYB, target of miR-142 and miR-150, were increased in *Hdac7^fl/−^* cells (Figure 5H). On the other hand, we found decreased expression of PUMA and IRF4, both targets of miR-125b, upon HDAC7 deficiency (Figure 5I). Globally, our data indicate that, through the regulation of *Tet2*, HDAC7 controls the expression levels of crucial miRNAs of the immune system.

### HDAC7 regulates 5-hmC levels and expression of LINE-1 elements

**Figure 6.**
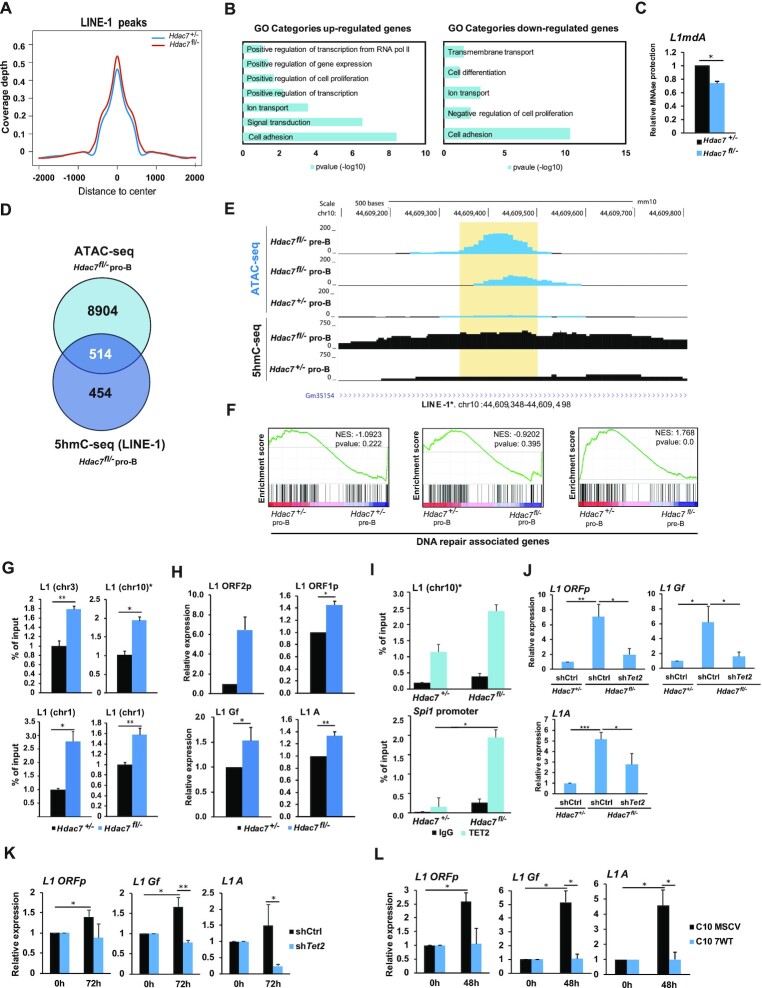
HDAC7 regulates 5-hydroxymethylation levels of transposable LINE-1 elements. (**A**) 5-hmC coverage depth (per base pair per peak per 10 million mapped reads) of 5-hmC peaks located in LINE-1 elements (−2 kb to +2 kb) in wild-type or HDAC7-deficient pro-B cells. (**B**) Gene Ontology (GO) enrichment analysis of genes associated to up-regulated (left panel) and down-regulated (right panel) regions in HDAC7-deficient pro-B cells respect to control pro-B cells in hMeDIP-seq shown in (A). (**C**) RT-qPCR showing protection of MNase-digested DNA at the 5min time point in wild-type or HDAC7-deficient B cells. (**D**) Venn diagram comparing enriched peaks associated genes in ATAC-seq from *Hdac7^fl/−^* pro-B cells and LINE-1 enriched peaks associated genes in 5hmC-seq (or hMeDIP-seq) in *Hdac7^fl/−^* pro-B cells compared to control cells. (**E**) Example of 5-hmC and ATAC-seq enrichment in young retrotransposon (L1) from peaks detected in hMeDIP-seq and ATAC-seq data. The peak location found common in the two omics analyses is located in the orange-shaded rectangle. (**F**) GSEA analysis comparing wild-type pre-B or HDAC7-deficient pro-B and pre-B cells to wild-type pro-B cells in expression profiles related to DNA repair signatures. DNA repair dataset was retrieved from www.gsea-msigdb.org. (**G**) hMeDIP-qPCR analysis of 5-hmC enrichment at LINE-1 retrotransposable elements in wild-type or HDAC7-deficient pro-B cells. (**H**) RT-qPCR analysis of the expression of proteins encoded by LINE-1 (ORFp1, ORFp2) and the most active L1 elements subfamilies (Gf, A) in wild-type or HDAC7-deficient pro-B cells. (**I**) ChIP-qPCR analysis of TET2 recruitment at hydroxymethylated L1 elements. TET2 enrichment at the *Spi1* promoter was used as a positive control. (**J–L**) As in Figure 4H-J, but analyzing the expression levels of L1 associated regions (L1 ORFp, L1 Gf and L1A). Data from (**G–L**) are represented as mean ± SEM of *n* = 3. *P < 0.05, **P < 0.01, ***P < 0.001, unpaired *t* test.

Our hMeDIP-seq approach also revealed that, according to the average signal from all the peaks obtained, the signal intensity of 5-hmC peaks associated with LINE-1 elements in HDAC7-deficient pro-B cells was higher than in their wild-type counterparts (Figure 6A). Gene ontology analysis of genes located in regions associated to LINE-1 elements revealed that 5-hmC enriched genes in *Hdac7^fl/^^−^* pro-B cells were more associated to gene activation and cell proliferation, whereas down-regulated LINE-1-related genes were involved in negative regulation of cell proliferation and cell differentiation (Figure 6B). In addition, we found that specific LINE-1-related loci were more susceptible to MNAse treatment in HDAC7-deficient B cells than wild-type cells and, therefore, more predisposed for aberrant upregulation (Figure 6C). Accordingly, we found that >50% of LINE-1 related peaks (associated 514 to genes) with 5-hmC enrichment were located in more open chromatin regions (Figure 6D). Hence, HDAC7 depletion was correlated with increased chromatin accessibility and enhanced or uncontrolled gene activation in LINE-1 associated loci (see examples in Figure 6E and Supplementary Figure S6A). Aberrant expression of LINE-1 elements is associated with genome instability. Accordingly, GSEA analysis of our RNA-seq data showed that the gene signatures in HDAC7-deficient pro-B or pre-B cells were associated to impaired DNA repair mechanisms (Figure 6F), suggesting a potential increase of genomic instability in the absence of HDAC7 that could correlate with LINE-1 aberrant expression. In fact, genes included in DNA repair geneset (form GSEA) that were down-regulated in HDAC7-deficient pro-B cells were related to DNA damage response and DNA repair abilities (Supplementary Figure S6B, C), supporting the potential affection of DNA repair capacity upon HDAC7 deficiency. Regions with differential 5-hmC peaks were validated by hMeDIP-qPCR assays, confirming that the absence of HDAC7 from pro-B cells resulted in higher levels of 5-hmC in LINE-1 elements (Figure 6G). Moreover, we observed a significant increase in the expression of LINE-1 transcripts from the most active families in HDAC7-deficient pro-B cells (Figure 6H), reinforcing the correlation between 5-hmC enrichment and gene activation. Previously published data revealed that TET1 and TET2 are recruited to the 5′ UTR of young LINE-1 elements in embryonic stem cells (54). We confirmed by ChIP-qPCR that TET2 was recruited to LINE-1 elements with enhanced 5-hmC in HDAC7-deficient pro-B cells; TET2 recruitment to the *Spi1* promoter was used as a positive control (Figure 6I). Finally, using samples from the gain and loss of function experimental approaches shown in Figure 4H–J, we further demonstrated that HDAC7-mediated LINE-1 regulation depends on *Tet2* expression (Figure 6J-L). Overall, our data indicated that HDAC7 plays a role in preserving the chromatin state and genome integrity in B cells by restricting the expression levels of TET2, which consequently leads to the maintenance of physiological levels of 5-hmC at retrotransposon elements.

## DISCUSSION

Here we reveal an unprecedented HDAC7-mediated molecular mechanism that preserves the correct chromatin conformation, histone marks deposition and DNA 5-hydroxymethylation state. Notably, this state is essential for B cell identity and, consequently, for a correct gene expression pattern during early B cell development. HDAC7 deficiency resulted in a global chromatin de-compaction that significantly increased its accessibility. This correlated with a global increased of H3K27ac in the absence of HDAC7 from pro-B cells. Chromatin organization is dynamically reshaped during B cell development, obtaining unique populations at each differentiation stage (55); however, this process is highly controlled, and alterations in chromatin state (such as that observed here in HDAC7-deficient cells) can alter the transcriptional regulation and gene expression patterns, which can drive malignant transformation (56). In line with the increased chromatin accessibility, HDAC7 deficiency also caused a significant decrease in H3K9me3, a hallmark of heterochromatin state, which is involved in maintaining lineage stability and preventing cell reprogramming (47,57,58). TET enzymes are mainly recruited to open chromatin regions; therefore, euchromatin (unlike heterochromatin) is enriched in 5-hmC (49). HDAC7 deficiency results in a global decrease of heterochromatin regions and enhanced TET2 recruitment. It has been reported that TET2 loss at stem cell stages produced an aberrant number of myelomonocytic cells and impairment in the expression of macrophage markers such as Mac-1 in myeloid cells (20,22). Within the B cell lineage, TET2 conditional deficiency at pro-B cell stage does not cause any phenotype during development and differentiation. Only conditional deletion of both TET2 and TET3 lead to defective B cell development (52). Even though there is no phenotype observed by TET2 deficiency *in vivo*, TET2 has been reported to play a critical role in mediating the hydroxymethylation of cytosine residues from myeloid genes during pre-B cells conversion into macrophages (24,29). Despite loss of TET2 and TET3 leads to aberrant lymphocyte development and related disorders (59,60), and loss of TET2 enzymatic activity appears to mainly affect myeolopoiesis (23,60), our results strongly suggest that HDAC7 is a critical factor that preserves B cell identity and correct DNA hydroxymethylation state via *Tet2* gene silencing.

Previous studies have established a close relationship between transcription regulators and dynamic changes in DNA methylation during B cell development and commitment, specifically at the pro-B to pre-B cell transition (14,59). Hematopoietic cells present low hydroxymethylation levels (∼0.2%) compared to other cell types, such as Purkinje cells or embryonic stem cells (∼5%) (50,61). However, we observed a significant decrease of heterochromatin, and an increase in 5-hmC, upon HDAC7 deficiency, leading to several molecular and biological consequences. First, we found a high percentage of 5-hmC peaks located in intergenic and distal promoter regions. These results may imply that distal regulatory regions with enhancer features are dependent on TET2-mediated DNA demethylation and may correlate with the presence of additional mechanisms that control DNA methylation status at promoter-associated regions (62,63). *Jun* and *Fosl2* were found among the myeloid and T cell genes with increased 5-hmC levels in HDAC7-deficient B cells. *Fosl2* is a TET2-activated gene during the transdifferentiation of pre-B cells to macrophages (24), and *Jun* undergoes enhancer demethylation prior to B cell being reprogrammed into induced pluripotent stem cells (iPSCs) (64). The finding that lineage-inappropriate genes are already marked with 5-hmC in B cell progenitors supports the notion that they may be epigenetically poised in early stages of development.

HDAC7 deficiency also led to differential 5-hmC levels in some regions containing miRNAs. miRNAs are epigenetic players that have crucial roles in multiple developmental processes, and their de-regulation is involved in many biological disorders. Recent studies have demonstrated that miRNAs have a role in normal and malignant B cell development, by modulating the expression of key regulatory genes (65–67). For instance, miR-34a is strongly expressed in myeloid cells, and its constitutive expression in B cells blocks the pro-B to pre-B cell transition (26,68); notably, this is the biological effect that we observed upon HDAC7 deficiency. miR-150, which is down-regulated in pro-B cells from *Hdac*7-null mice, is related to B cell development and performs tumor-suppressor functions in leukemic cells. miR-142 and miR-181 are highly expressed in a cell-specific manner (69), such as in hematopoietic cells. Specifically, B cell function is impaired in miR-142 deficiency conditions (67), and miR181a regulates positively the B lymphocyte differentiation (70). Previous studies indicated that miR-126 is downregulated in lymphoid cells, (71,72), which is consistent with our results. Moreover, miR-29b is activated by C/EBPα and represses *Tet2* expression, which concords with C/EBPα and *Tet2* up-regulation when HDAC7 is deficient (73). miR-34a is strongly expressed in myeloid cells, and its constitutive expression in B cells blocks the pro-B to pre-B cell transition (68). Finally, some members of the miR-99 family, such as miR-99b, are abundant in macrophages, neutrophils, and monocytes. Here, we observed that another member of the family related to leukemic stem cells, miR-99a, was upregulated in HDAC7-deficient pro-B cells. Thus, our results demonstrate that HDAC7 can also exert its gene repressive function during early B cell development by regulating gene expression, presumably by interacting with its classical partner MEF2C, which may be recruited to miRNA regulatory regions, as it does at other specific miRNA regions in the skeletal muscle (74).

We have detected increased 5-hmC enrichment and a more open chromatin state in some regions containing LINE-1 transposable elements in HDAC7-deficient pro-B cells. LINE-1 elements are the only active autonomous retrotransposons in the mammalian genome and, consequently, a potential disturber of chromatin stability (75). In fact, LINE-1 transcripts from the A and Gf subfamilies, which present increased expression in HDAC7-deficient pro-B cells, contain some members that still have the full-length transcript, which maintains its retrotransposon activity in the mouse genome (76). The fact that increased 5-hmC is associated to TET2 recruitment suggests that HDAC7 might be required to preserve chromatin integrity by mediating the silenced status of LINE-1 elements. Of note, recent studies have shown that tight regulation of TET2 activity is essential for correct maintenance of genome stability: TET2 deficiency produces defects in DNA damage response, and its overexpression produces chromosome instability and aneuploidy due to a collapse in BER activity (77,78). Thus, results from this paper suggest that TET2 aberrant expression by HDAC7-deficient B cells may impair their capacity to repair DNA damage, which agree with the observed higher cell death rates in these cells that we reported in our previous published work (26).

Significant loss of H3K9me3 enrichment upon HDAC7 deficiency could also correlate with LINE-1 deregulated expression, since this heterochromatin mark is required to repress aberrant expression of retrotransposons in mammal embryonic stem cells (79,80). However, given that DNA methylation is the main source of LINE-1 repression in more differentiated cells, we suggest that LINE-1 are silenced due to DNA demethylation caused by TET2 upregulation; this would reinforce the effects of DNA methylation loss following TET2 up-regulation upon HDAC7 deficiency. This result indicates that LINE-1 deregulation is produced as a consequence of HDAC7 deficiency.

Our results represent a significant step forward in our understanding of how B cells acquire their genetic identity, from three different perspectives. First, we identified HDAC7 as a chromatin modulator that regulates the heterochromatin state and histone marks deposition in early B cell development. Second, we demonstrated that HDAC7 is the specific transcription repressor that controls TET2 activity, which it achieves by fine-tuning its physiological expression levels in pro-B cells. This may represent the mechanistic explanation for the different TET2 expression levels observed in myeloid and lymphoid cells. Third, our results reveal an unexpected role for HDAC7 in controlling proper DNA 5-hydroxymethylation status and expression of lineage- or functionally-inappropriate genes, microRNAs, and non-coding elements (such as LINE-1 elements) in pro-B cells. We recently identified HDAC7 to be a novel biomarker and prognostic factor in infants (<1-year-olds) with pro-B acute lymphoblastic leukemia (pro-B-ALL) and MLL-AF4 rearrangement (27). This subgroup of pediatric patients presents an extremely adverse outcome, with survival rate below 35%, and the loss of HDAC7 is associated with a worse prognosis. Therefore, the elucidation of the exact molecular mechanisms that HDAC7 exert during physiological early B cell development will be crucial to understand how their deregulation can result in B cell–associated malignancies, with potential implications in the clinics.

**Figure 7.**
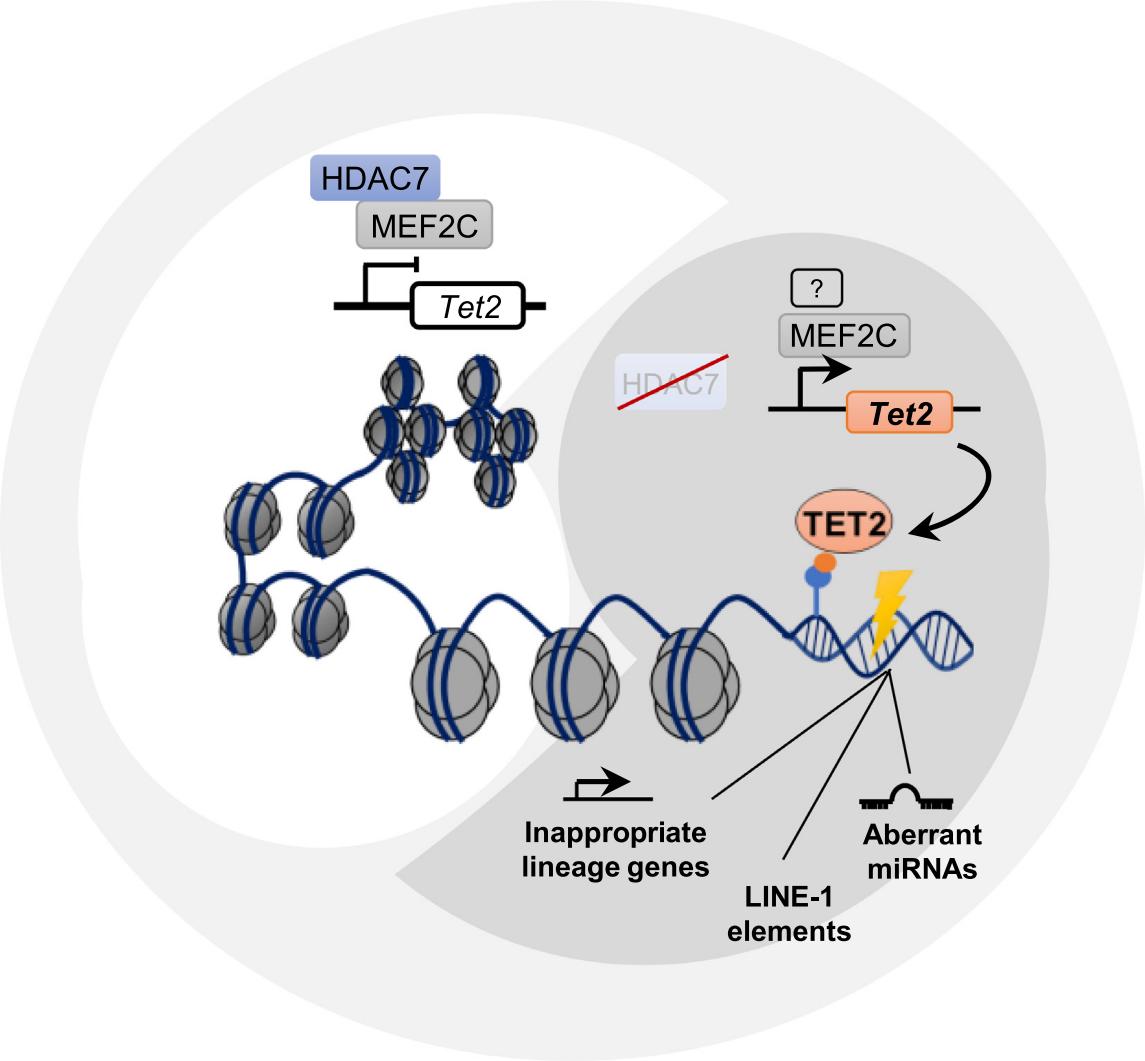
Visual representation summarizing the role of HDAC7–TET2 axis in the regulation of proper gene silencing in early B cell development.

Altogether, our findings lead us to a proposed model by which HDAC7 functions during early B cell development are not restricted to controlling expression by direct recruitment to its target genes. Rather, HDAC7 governs the expression of another crucial epigenetic regulator, TET2. The identified HDAC7–TET2 epigenetic axis is essential to preserve proper 5-hmC and histone marks levels, chromatin compaction, and expression of miRNAs and LINE-1 elements (Figure 7). We anticipate that our findings may open new avenues to understanding the consequences of HDAC7 deregulation in altering the molecular mechanism found in B cell–related malignancies, eventually leading to strategies to develop therapies for these pathologies.

## DATA AVAILABILITY

Data are available in GEO repository as follows: RNA-seq (GSE171855), ATAC-seq (GSE204672), ChIP-seq (GSE204673) and hMeDIP-seq (GSE135263).

## Supplementary Material

gkac619_Supplemental_File

## Notes

Present address: Maria Vila-Casadesús, Bioinformatics Platform, CIBER Hepatic and Digestive Diseases (CIBERehd), 08036 Barcelona, Catalonia, Spain.
