# Supplementary material for: The HDAC7–TET2 epigenetic axis is essential during early B lymphocyte development
Source: Nucleic Acids Res. 2022 Jul 29;50(15):8471–90. doi: 10.1093/nar/gkac619 (PMC9410891; doi:10.1093/nar/gkac619)
Supplement: gkac619_Supplemental_File [file gkac619_supplemental_file.pdf]

Supplementary Information

The HDAC7-TET2 epigenetic axis is essential during early B lymphocyte development

Alba Azagra, Ainara Meler, Oriol de Barrios, Laureano Tomás-Daza, Olga Collazo, Beatriz Monterde, Mireia Obiols, Llorenç Rovirosa, Maria Vila-Casadesús, Mónica Cabrera-Pasadas, Mar Gusi-Vives, Thomas Graf, Ignacio Varela, José Luis Sardina, Biola M Javierre, and Maribel Parra

Supplementary Figure Legends

Supplementary Figure 1. HDAC7 deficiency results in global chromatin de-condensation and increase 5-hmC levels

(**A**) Flow cytometry plots showing the gating strategy to sort pro-B (IgM$^-$, CD19$^+$, B220$^-$, CD43$^+$) and pre-B cells (IgM$^-$, CD19$^+$, B220$^-$, CD43$^-$) from the bone marrow of control and *Hdac7*$^{fl/-}$ conditional mice. (**B**) Schematic representation of up-regulated and down-regulated genes between different cell types and conditions of the RNA sequencing experiment in Figures 1B-E. (**C**) Heat map showing most down-regulated genes in *Hdac7*$^{fl/-}$ pro-B and pre-B cells compared to control cells (left panel). Absolute expression of *Igkv4-40*, *Igkv4-55* and *Igkv4-57-1* genes in HDAC7-deficient pro-B and pre-B cells *versus* control counterpart cells, using data from RNA-seq (right panel). Quantification values shown are expressed in FPKM values. (**D**) Gene ontology (GO) analysis of down-regulated genes in HDAC7 deficient pro-B and pre-B cells versus control pro-B cells. (**E**) BP ontology analysis of up-regulated genes in HDAC7-deficient pro-B and pre-B cells compared to control pro-B cells. (**F**) MF ontology analysis of up-regulated genes in HDAC7-deficient pro-B and pre-B cells compared to control pro-B cells. MF and BP correspond to Molecular Function and Biological Process, respectively.

Supplementary Figure 2. HDAC7 controls DNA 5-hmC through *Tet2* regulation in pro-B and pre-B cells.

(A) *Tet1* and *Tet3* expression profiles in hematopoietic cells subsets. Data were obtained from the Immunological Genome Project (Immgen) database. (**B**) Heat map showing the expression of *Hdac7, Tet1, Tet2 and Tet3* in different hematopoietic cell types. Data was obtained from Immgen database using “My Gene set” tool. (**C**) Analysis by RT-qPCR of *Tet1, Tet2 and Tet3* mRNA levels in bone marrow from wild-type and HDAC7-deficient pro-B cells and in Cd11b$^+$ cells. (**D**) RNA-seq signal profiles for *Pax5* gene of HDAC7-deficient pro-B and pre-B cells, and control pro-B cells. Orange shadow highlights *Pax5* promoter region. (**E**) Analysis by RT-qPCR of *E2a* and *Pax5* genes in pro-B and pre-B cells from wild-type and HDAC7-deficient mice. (**F**) As in (**E**), but for *Hdac4*, *Hdac5* and *Hdac9* genes. Data from panels C, E and F are represented as mean of n=3 ± SEM; unpaired t test were used to determine significance (*p<0.05, **p<0.01).

Supplementary Figure 3. *Tet2* is a direct HDAC7 target gene in pro-B and pre-B cells

(A) ATAC-seq signal profile of HDAC7-deficient and control pro-B cells over the transcription factor *Pax5* genomic sequence. (**B**) Venn Diagram comparing genes enriched in H3K9/K14ac ChIP-seq epigenetic mark and genes detected as open regions by ATAC-seq in *Hdac7*$^{fl/–}$ pro-B cells compared to control pro-B cells. Tet2 is included among the subset of overlapping genes. (**C**) Depth coverage of H3K27ac peaks signal in all regions from ChIP-sequencing experiments in Figures 3D-E. (**D**) Genomic distribution of H3K27ac enriched peaks in *Hdac7*$^{+/–}$ and *Hdac7*$^{fl/–}$ pro-B cells determined by ChIP-seq experiments in Figures 3D-E. (**E**) Venn Diagram comparing genes enriched in H3K27ac epigenetic mark and genes detected as open regions by ATAC-seq in *Hdac7*$^{fl/–}$ pro-B cells compared to control pro-B cells. (**F**) Depth coverage of H3K27me3 peaks signal in all regions from ChIP-sequencing experiments in Figures 3F-G. (**G**) As in (D), but with gene regions enriched for H3K27me3 epigenetic mark. (**H**) Venn Diagram comparing genes enriched in H3K27me3 epigenetic mark from wild-type pro-B cells and genes detected as open regions by ATAC-seq in *Hdac7*$^{fl/–}$ pro-B cells compared to control pro-B cells. (**H**) H3K27ac and H3K27me3 ChIP-seq signal profile of HDAC7-deficient and control pro-B cells over the transcription factor

Pax5 genomic sequence. (**I**) ChIP-qPCR data after immunoprecipitation with H3K9me3 antibody. Enrichment of *Tet2* promoter (left panel) and *Tet2* enhancer (right panel) regions is quantified as % of input. Data is represented as mean ± SEM of n = 3. *p < 0.05, unpaired T-test.

Supplementary Figure 4. HDAC7 deficiency results in increased recruitment of TET2 and altered 5-hmC enrichment at B cell lineage inappropriate genes
(A) Depth coverage of 5-hmC peaks signal in intergenic and promoter (TSS) regions from 5-hmC-sequencing experiments in Figures 4A-C. (**B**) Genome browser snapshot of 5-hmC peaks, ATAC-seq peaks and RNA-seq peaks at *FosB* promoter in wild-type pro-B and HDAC7-deficient pro-B and pre-B cells. (**C**) Analysis by RT-qPCR of inappropriate lineages genes *FosL2* and *FosB in* wild-type pro-B cells and HDAC7-deficient pro-B and pre-B cells. (**D**) RNA-seq signal profile of HDAC7-deficient pro-B and pre-B and control pro-B cells of *Itgb2* and *Cd69* genes. (**E**) Analysis of TET2 recruitment at other targets such as *Fosl2, Ahnak and Itgb2* genes by ChIP-qPCR, quantified as % of input. (**F**) Schematic representation of the three experimental approaches used in gain and loss of function experiments in Figures 4H-J. Approach 1 uses primary pro-B cells from *Hdac7*$^{+/-}$ and *Hdac7*$^{fl/-}$ mice and approaches 2 and 3 use pre-B cell lines that transdifferentiate into macrophages upon β-estradiol treatment. Blue lines indicate the effects caused by the inhibition of *Tet2* expression through sh*Tet2* retroviral infection or HDAC7 induction by MSCV-7WT infection. TF=transcription factor. Data in panel (D) is represented as mean ± SEM of n = 3. *p < 0.05, unpaired T-test.

Supplementary Figure 5. HDAC7 directs B cell–specific miRNA hydroxymethylation and expression patterns
(A) Example of 5-hmC and open chromatin peaks enriched at aberrant miRNAs such as miR-29b and miR-99a from hMeDIP-seq and ATAC-seq experiments. **(B)** Example of 5-hmC and open chromatin enriched peaks at B-cell related miRNAs such as miR-150 and miR-181a from hMeDIP-seq and ATAC-seq experiments.

Supplementary Figure 6. HDAC7 regulates 5-hydroxymethylation levels of transposable LINE-1 elements
(A) Example of 5-hmC and open chromatin peaks enriched at LINE-1 associated regions from hMeDIP-seq and ATAC-seq experiments. (**B**) GO analysis of genes included in DNA repair geneset (from GSEA) that are down-regulated in *Hdac7*$^{fl/-}$ pro-B cells. (**C**) Table showing genes lists included in clusters from GO analysis from panel (B).

Table S1. Primer sequences used for RT-qPCR and ChIP qPCR experiments.
Table S2. Relative expression values of microRNAs showing differential expression profiles in control and *Hdac7*$^{fl/-}$ pro-B cells.

Supplementary Figure 1

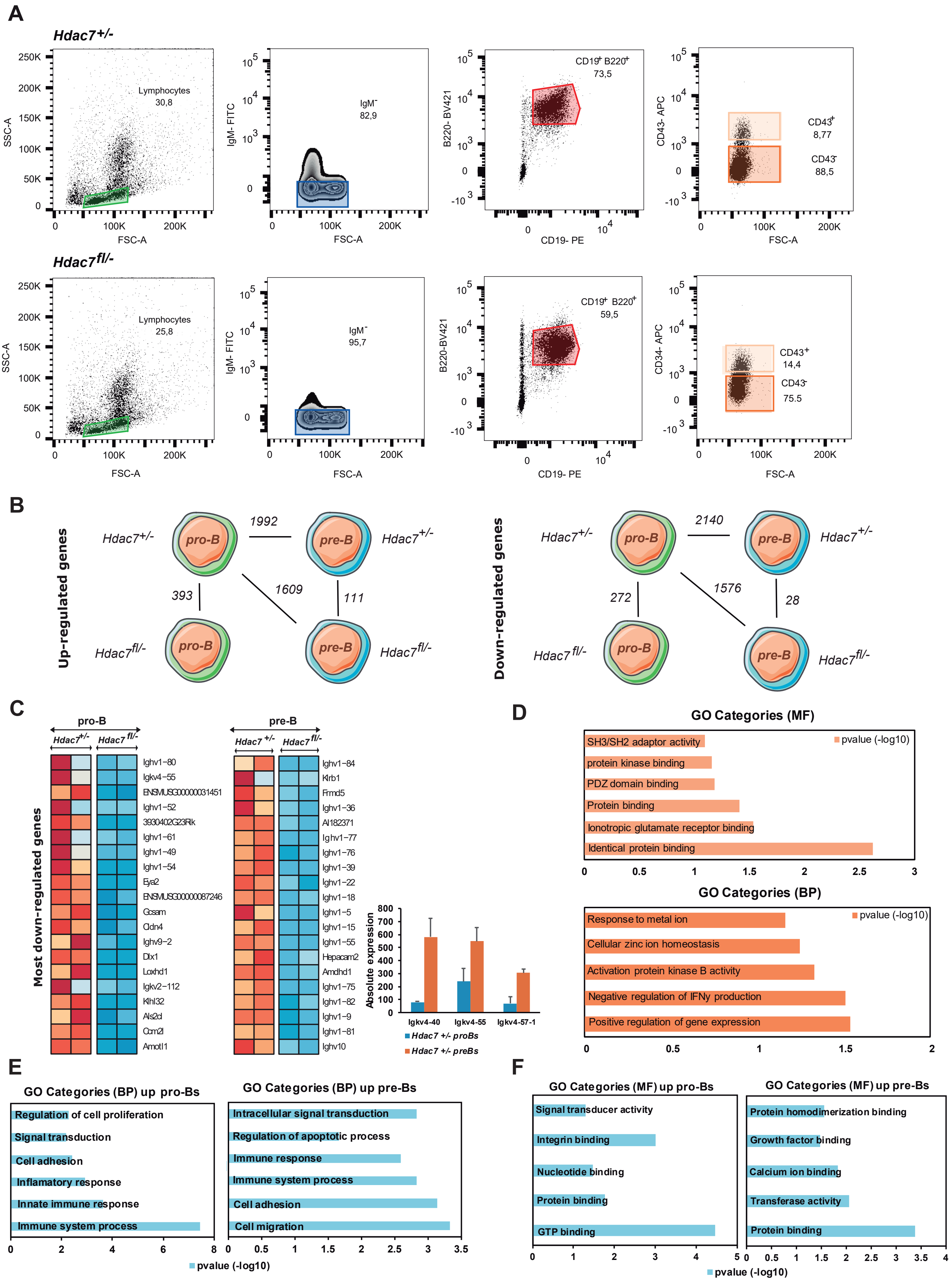

Supplementary Figure 2

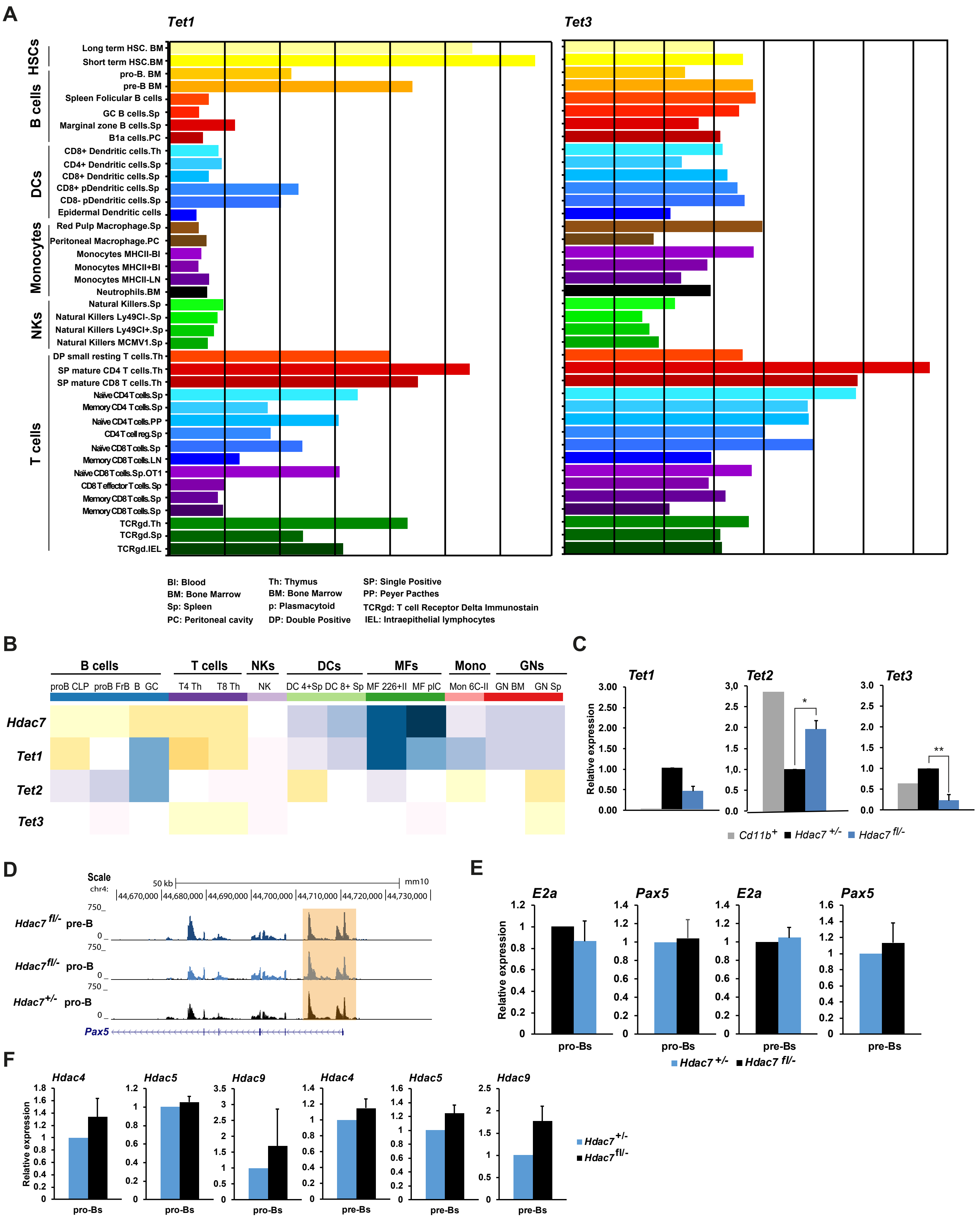

Supplementary figure 3

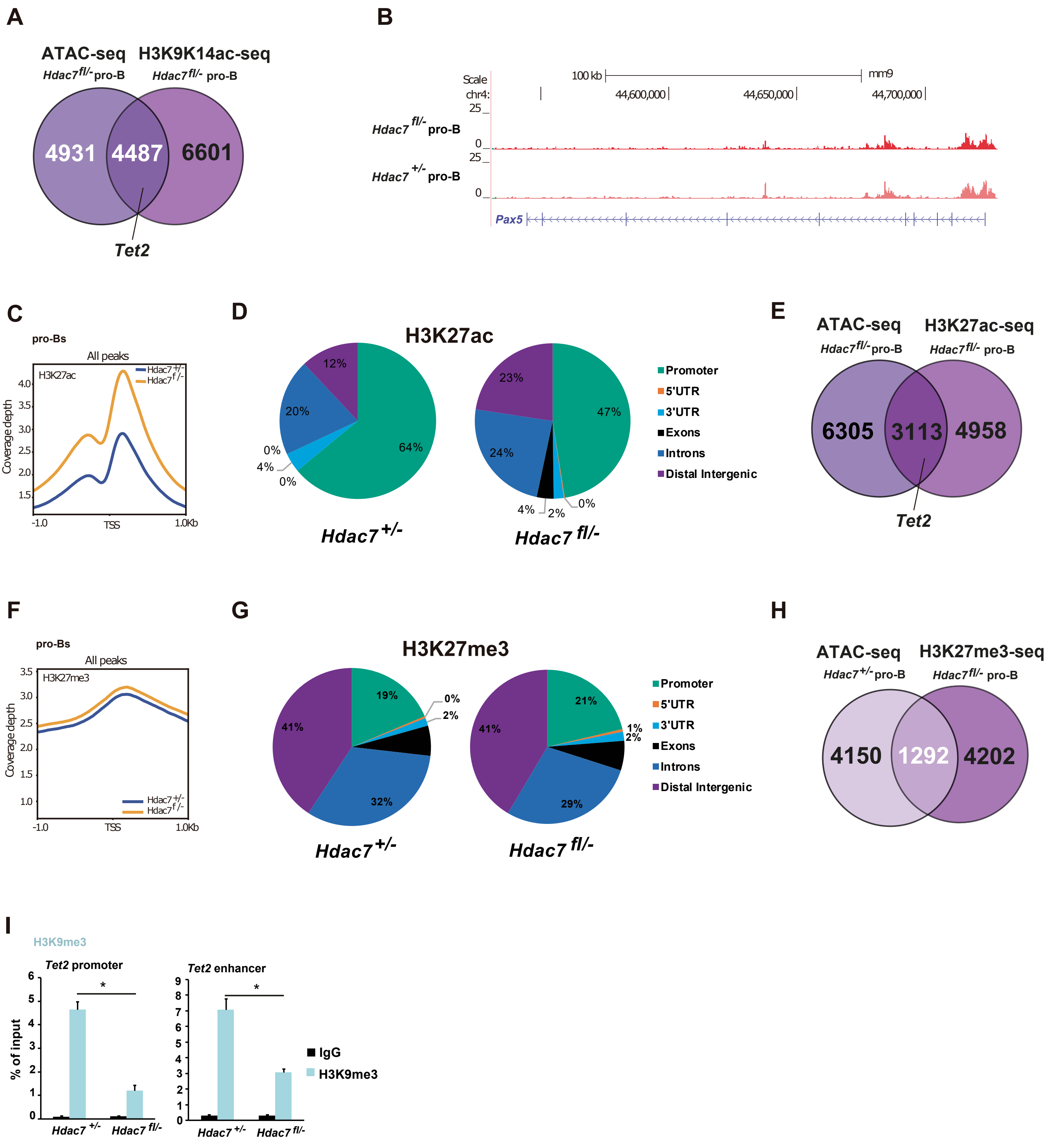

Supplementary Figure 4

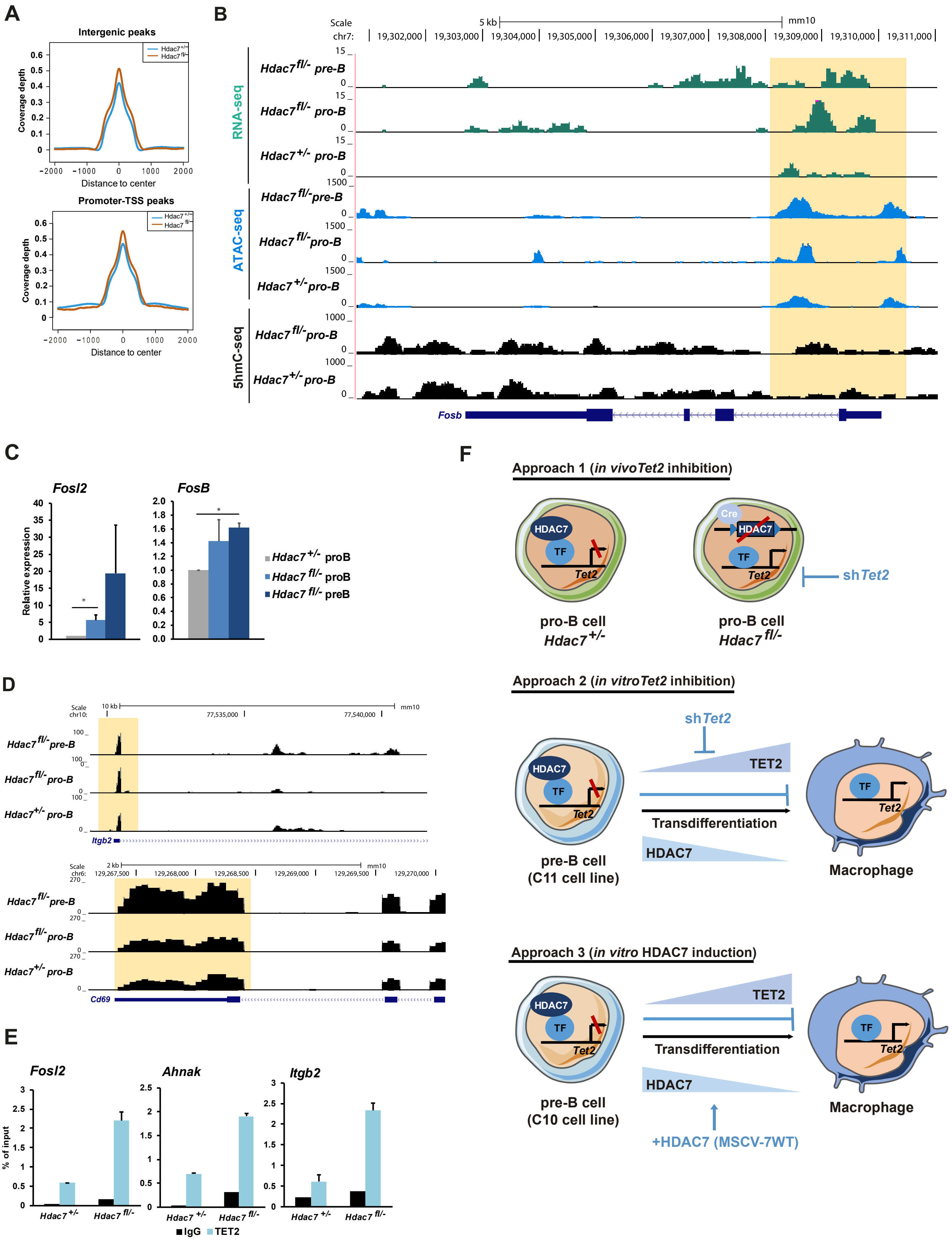

Supplementary Figure 5

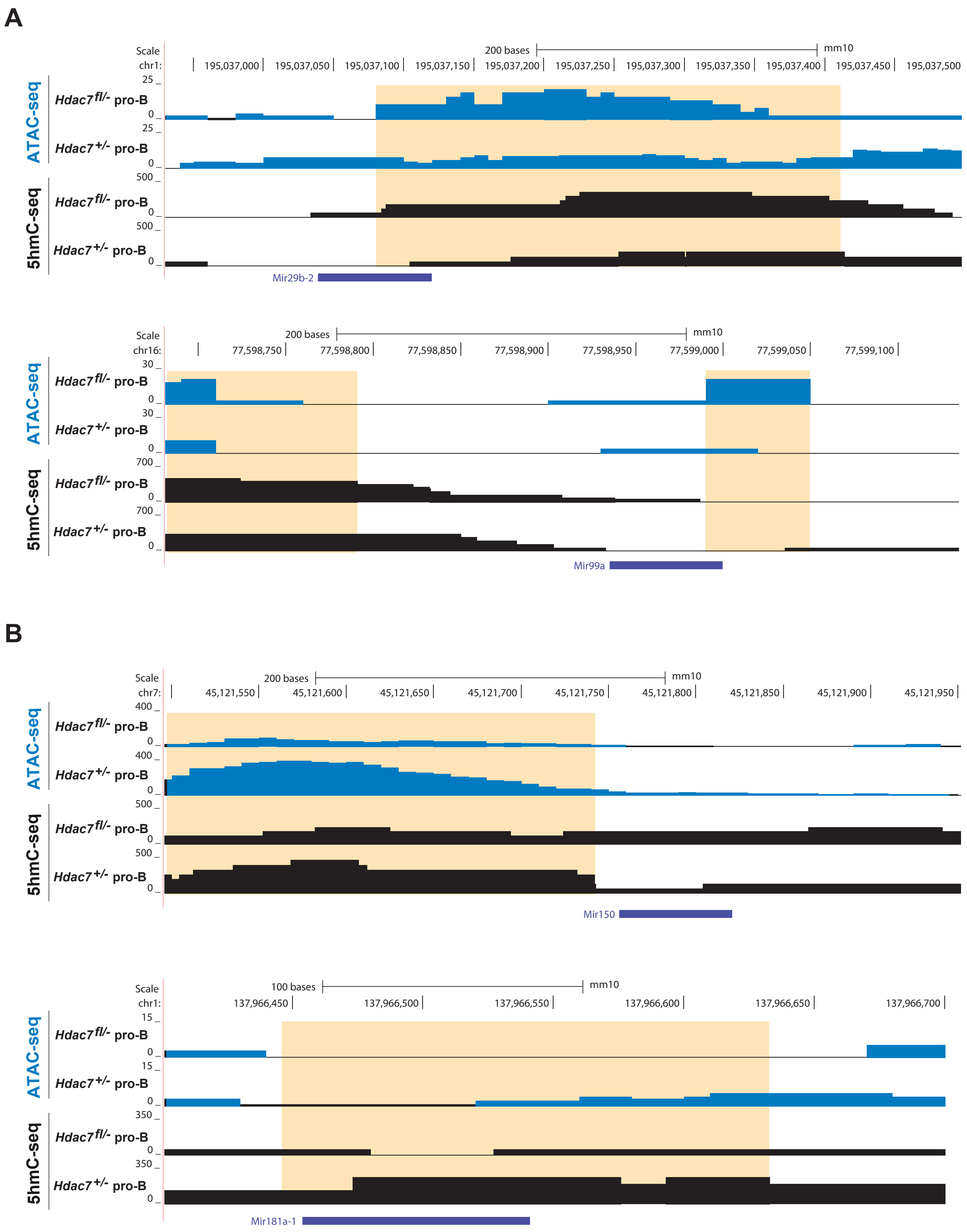

Supplementary Figure 6

A

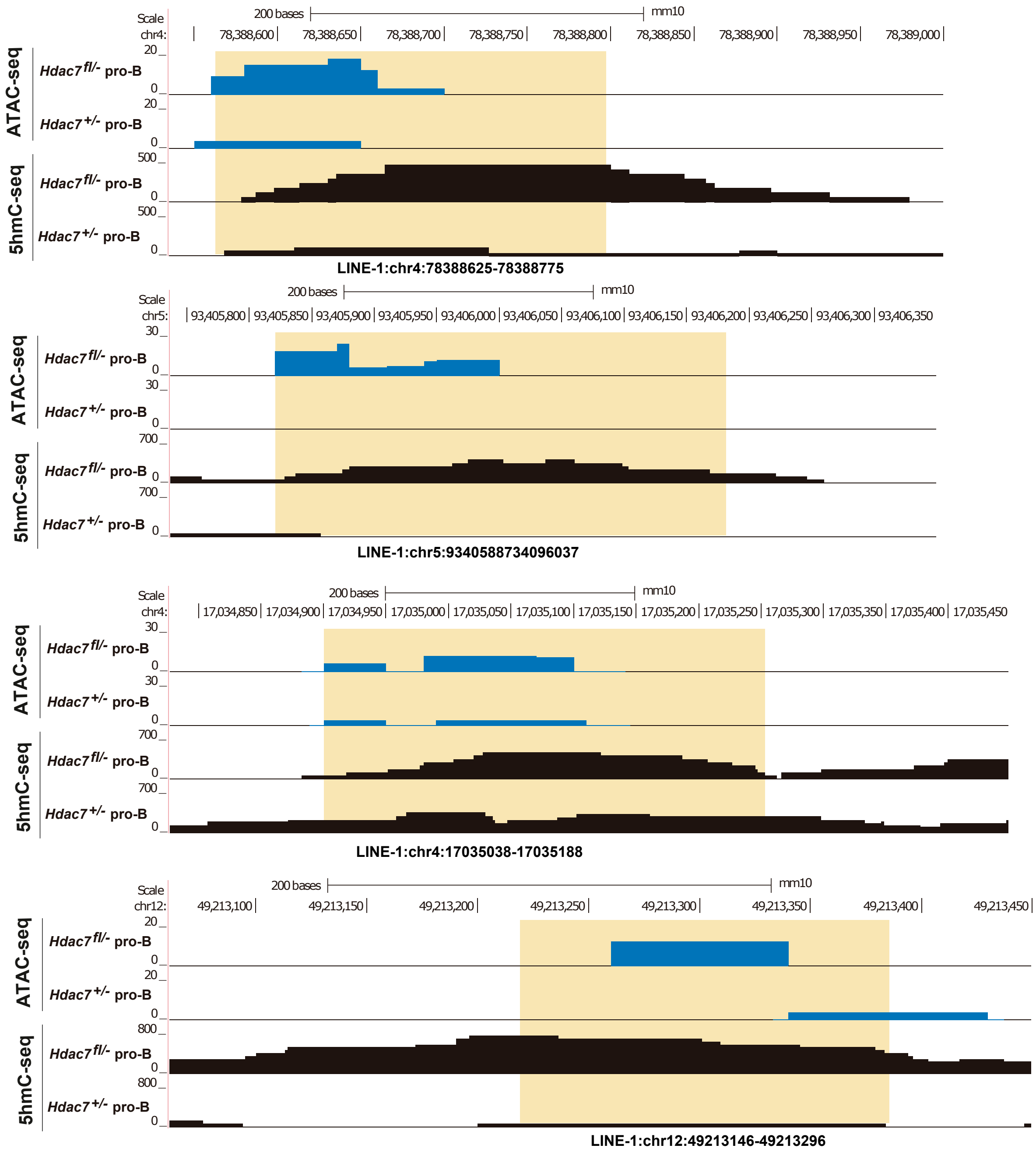

B

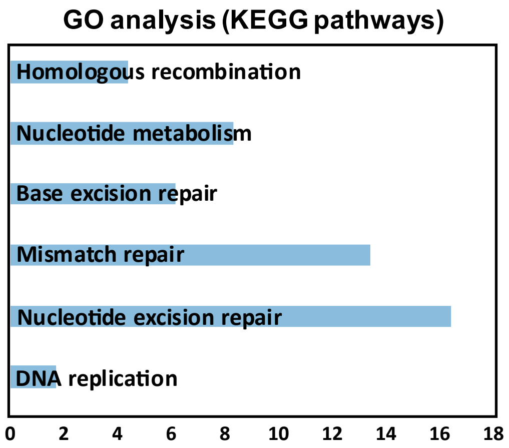

C

Nucleotide excision repair	Mismatch repair	Base excision repair	Cellular response to DNA damage
DDB1	LIG1	FEN1	RAD51
ERCC2	POLD1/3	LIG1	DDB1
ERCC8	PCNA	POLD1/3	ERCC2/5
GTF2H1/3	RCF3/4/5	POLL	FEN1
PCNA	RPA2/3	PCNA	GTF2H1/3
LIG1			POLD1
POLD1/3			POLL
RFC3/4/5			POLH
RPA2/3			PCNA
			SSRP1

Table S1- primer sequences

Name	Sequence	Purpose	Source
Tet1 RTqPCR Fw	ACAAGCAGATGGCTCCAGTT	Real time qPCR	Kallin et al, Moll Cell 2012
Tet1 RTqPCR Rv	GCAACAGGTGACACCAGAGA	Real time qPCR	Kallin et al, Moll Cell 2012
Tet2 RTqPCR Fw	ATCCAAACCGAAGCTGAATG	Real time qPCR	Kallin et al, Moll Cell 2012
Tet2 RTqPCR Rv	CTTGCCCTACCACCGTTTTA	Real time qPCR	Kallin et al, Moll Cell 2012
Tet3 RTqPCR Fw	TCCGGATTGAGAAGGTCATC	Real time qPCR	Kallin et al, Moll Cell 2012
Tet3 RTqPCR Rv	TCCTCCAGTGTGTGTCTTCG	Real time qPCR	Kallin et al, Moll Cell 2012
Pax5 RTqPCR Fw	GCCTGGGAGTGAATTTTCTGGA	Real time qPCR	This paper
Pax5 RTqPCR Rv	GGGCTGCAGGGCTGTAATAGTAT	Real time qPCR	This paper
E2a RTqPCR Fw	AGGAAATCGCATCAGTAGCC	Real time qPCR	This paper
E2a RTqPCR Rv	AGGGACAGCACCTCATCTGT	Real time qPCR	This paper
Gapdh RTqPCR Fw	ACATCTCACTCAAGATTGTCAGCA	Real time qPCR	Dawlaty et al, Develop Cell 2014
Gapdh RTqPCR Rv	ATGGCATGGACTGTGGTCAT	Real time qPCR	Dawlaty et al, Develop Cell 2014
FosL2 RTqPCR Fw	TGGAGTGATCAAGACCATCG	Real time qPCR	Kallin et al, Moll Cell 2012
FosL2 RTqPCR Rv	GTTTCTCTCCCTCCGGATTC	Real time qPCR	Kallin et al, Moll Cell 2012
Jun RTqPCR Fw	ACGACCTTCTACGACGATGC	Real time qPCR	Kim et al, PNAS 2013
Jun RTqPCR Rv	CCAGGTTCAAGGTCATGCTC	Real time qPCR	Kim et al, PNAS 2013
L1-ORFp1 RTqPCR Fw	ACTCAAAGCGAGGCAACACTAGA	Real time qPCR	de la Rica et al, Genome Biol 2016
L1-ORFp1 RTqPCR Rv	GTTCCAGATTTCTTTCCTAGGGTTTC	Real time qPCR	de la Rica et al, Genome Biol 2016
L1MdGf RTqPCR Fw	TGGAATACAGAGTGCCAGCC	Real time qPCR	de la Rica et al, Genome Biol 2016
L1MdGf RTqPCR Rv	GTGCTCTCACCAGGAAGGTG	Real time qPCR	de la Rica et al, Genome Biol 2016
L1MdA RTqPCR Fw	TCTGGTGAGTGGAACACAGC	Real time qPCR	de la Rica et al, Genome Biol 2016
L1MdA RTqPCR Rv	AGTCTCGAGTGGAGCGGAAG	Real time qPCR	de la Rica et al, Genome Biol 2016
miR-125b-5p Fw	GCAGTCCCTGAGACCCT	Real time qPCR	miRprimer (https://sourceforge.net/projects/mirprimer/)
miR-125b-5p Rv	CGAGTTTTTTTTTTTTTTTCACAAGT	Real time qPCR	miRprimer software (same source)
miR-34a-5p Fw	GCAGTGGCAGTGTCTTAG	Real time qPCR	miRprimer software (same source)
miR-34a-5p Rv	GGTCCAGTTTTTTTTTTTTTTTACAAC	Real time qPCR	miRprimer software (same source)
miR-28a Fw	CAGAAGGAGCTCACAGTCT	Real time qPCR	miRprimer software (same source)
miR-28a Rv	GGTCCAGTTTTTTTTTTTTTTTCTCA	Real time qPCR	miRprimer software (same source)
miR-150-5p Fw	CTCCCAACCCTTGTACCA	Real time qPCR	miRprimer software (same source)
miR-150-5p Rv	GGTCCAGTTTTTTTTTTTTTTTCACT	Real time qPCR	miRprimer software (same source)
miR-142-3p Fw	CGAGTGTAGTGTTTCCT	Real time qPCR	miRprimer software (same source)
miR-142-3p Rv	GGTCCAGTTTTTTTTTTTTTTTCCA	Real time qPCR	miRprimer software (same source)
miR-181a-5p Fw	CATTCAACGCTGTCGGT	Real time qPCR	miRprimer software (same source)
miR-181a-5p Rv	GGTCCAGTTTTTTTTTTTTTTTACTCA	Real time qPCR	miRprimer software (same source)
Hdac4 Fw	GCCATCTGTGATGCTTCTGA	Real time qPCR	This paper
Hdac4 Rw	ATTGGCATTGGGTCTCTGAT	Real time qPCR	This paper
Hdac5 Fw	AGCCATGGGATTCTGCTTCT	Real time qPCR	This paper
Hdac5 Rw	AGTCCACGATGAGGACCTTG	Real time qPCR	This paper
Hdac9 Fw	CCCCTATGGGAGATGTTGAG	Real time qPCR	This paper
Hdac9 Rw	CAATGCATCAAATCCAGCAG	Real time qPCR	This paper
Tet2-promoter ChIP Fw	CAAGCTTGAGGTCTGGGAGAA	ChIP-qPCR	This paper
Tet2-promoter ChIP Rv	CAGTCAGGCTGCTATCGAGT	ChIP-qPCR	This paper
Tet2-enhancer ChIP Fw	CTGAGAGCATCTCCCAGGTC	ChIP-qPCR	This paper
Tet2-enhancer ChIP Rv	GGAGTGAGGCAATACCAGGA	ChIP-qPCR	This paper

Table S1- primer sequences

L1-chr10-ChIP Fw	GTTGACCCCGTGTCAGTCTT	ChIP-qPCR	This paper
L1-chr10-ChIP Rv	GGCGACCTCAACTGAATGAT	ChIP-qPCR	This paper
Spi1 promoter ChIP Fw	GTAGCGCAAGAGATTTATGCAAAC	ChIP-qPCR	This paper
Spi1 promoter ChIP Rv	GCACAAGTTCCTGATTTTATCGAA	ChIP-qPCR	This paper
miR-125b-Tet2-ChIP Fw	TTCCCAAGCTGTCCGTTTAC	ChIP-qPCR	This paper
miR-125b-Tet2-ChIP Rv	TGGTGGTTTATGCCGAGAAT	ChIP-qPCR	This paper
miR-34a-Tet2-ChIP Fw	AGCCTCTCCATCTTCCTGTG	ChIP-qPCR	This paper
miR-34a-Tet2-ChIP Rv	CGTTGCTGACCTCTGACCTT	ChIP-qPCR	This paper
Jun ChIP Fw	GAGGTTGGGGGCTACTTTTC	ChIP-qPCR	This paper
Jun ChIP Rv	TGTCCTGCCCAGTGTTTGTA	ChIP-qPCR	This paper
Itgb2 ChIP Fw	CTACAAGCCCCTCCCTCTCT	ChIP-qPCR	Kallin et al, Moll Cell 2012
Itgb2 ChIP Rv	CCCAGGAGGAAGTTGAGTGA	ChIP-qPCR	Kallin et al, Moll Cell 2012
Jun 5hmC Fw	AAAGTCTGCCGGCCAATAG	hMeDIP-qPCR	This paper
Jun 5hmC Rv	GAACTTGACTGGTTGCGACA	hMeDIP-qPCR	This paper
Fosl2 5hmC Fw	GGAGCTTGCAGAGCAGAAAC	hMeDIP-qPCR	Kallin et al, Moll Cell 2012
Fosl2 5hmC Rv	CCTAACCAAGGCAGACAGGA	hMeDIP-qPCR	Kallin et al, Moll Cell 2012
L1 chr3 5hmC Fw	TTGTGCTTTTTCTTGGGCTA	hMeDIP-qPCR	This paper
L1 chr3 5hmC Rv	AGTTTTCCCTCCCTCTGCTC	hMeDIP-qPCR	This paper
L1 chr10 5hmC Fw	AAACCCACAGAATTGGAACG	hMeDIP-qPCR	This paper
L1 chr10 5hmC Rv	CCTCGTCTTTTCCTTCACTTTG	hMeDIP-qPCR	This paper
L1 chr1 5hmC Fw	AGCCTCCTTGATGTTCCTCTT	hMeDIP-qPCR	This paper
L1 chr1 5hmC Rv	TGAGAGCACAAACACTAAGCAA	hMeDIP-qPCR	This paper
β-globin Fw	AAGGTATGAGAATCCAGGCAG	MNAse, qPCR	Vazquez et al, Nucleic Acids Res 2019
β-globin Rv	GCCAAAACAATCACCAGCAC	MNAse, qPCR	Vazquez et al, Nucleic Acids Res 2019
L1mdA Fw	CAAACCCCTTCCACTCCACTCGAGC	MNAse, qPCR	Vazquez et al, Nucleic Acids Res 2019
L1mdA Rv	CCTTTCGCCATCTGGTAATC	MNAse, qPCR	Vazquez et al, Nucleic Acids Res 2019

Table S2. Relative expression values of microRNAs showing differential expression profiles in control and *Hdac7*$^{fl/-}$ pro-B cells.

miRNA Name	Wt1	Wt2	Ko1	Ko2	Mean Wt	Mean Ko	FC	Log2FC
mmu-miR-141-3p	0.03518	0.05	0.01914	0.02	0.04394	0.02011	2.18	1.13
mmu-miR-150-5p	3.95758	6.91	1.27930	1.42	5.43628	1.34796	4.03	2.01
mmu-miR-503-5p	3.14987	0.02	0.15820	0.00	1.58459	0.07981	19.85	4.31
mmu-miR-125p-5p	0.00243	0.01	0.02324	0.06	0.00708	0.04114	0.22	-2.18
mmu-mi125b-5pR-	0.00776	0.00	0.05818	0.08	0.00505	0.07002	0.07	-3.79
mmu-miR-126a-3p	0.01418	0.04	0.07275	0.17	0.02514	0.12053	0.21	-2.26
mmu-miR-196b-5p	0.00134	0.00	0.00777	0.02	0.00102	0.01587	0.06	-3.96
mmu-miR-214-3p	0.00050	0.00	0.01465	0.00	0.00047	0.00868	0.05	-4.20
mmu-miR-29b-5p	0.31845	0.55	1.93672	1.63	0.43660	1.78306	0.24	-2.03
mmu-miR-337-3p	0.00112	0.00	0.00075	0.03	0.00134	0.01552	0.09	-3.54
mmu-miR-34a-5p	0.00643	0.02	0.33496	0.13	0.01142	0.23492	0.05	-4.36
mmu-miR-465c-5p	0.00044	0.00	0.03096	0.07	0.00032	0.04874	0.01	-6.77
mmu-miR-466d-3p	0.01122	0.02	0.12842	0.35	0.01366	0.24153	0.07	-3.81
mmu-miR-667-3p	0.00199	0.00	0.00598	0.01	0.00166	0.00769	0.22	-2.21
mmu-miR-760-3p	0.00337	0.00	0.02158	0.02	0.00265	0.02143	0.12	-3.02
mmu-miR-99a-5p	0.00645	0.00	0.04043	0.07	0.00417	0.05587	0.07	-3.75
